# The ITS1-5.8S-ITS2 Sequence Region in the Musaceae: Structure, Diversity and Use in Molecular Phylogeny

**DOI:** 10.1371/journal.pone.0017863

**Published:** 2011-03-22

**Authors:** Eva Hřibová, Jana Čížková, Pavla Christelová, Stefan Taudien, Edmond de Langhe, Jaroslav Doležel

**Affiliations:** 1 Centre of the Region Haná for Biotechnological and Agricultural Research, Institute of Experimental Botany, Olomouc, Czech Republic; 2 Genome Analysis, Leibniz Institute for Age Research, Fritz Lipmann Institute (FLI), Jena, Germany; 3 Laboratory of Tropical Crop Improvement, Katholieke Universiteit Leuven, Leuven, Belgium; Institut de Biologia Evolutiva - Universitat Pompeu Fabra, Spain

## Abstract

Genes coding for 45S ribosomal RNA are organized in tandem arrays of up to several thousand copies and contain 18S, 5.8S and 26S rRNA units separated by internal transcribed spacers ITS1 and ITS2. While the rRNA units are evolutionary conserved, ITS show high level of interspecific divergence and have been used frequently in genetic diversity and phylogenetic studies. In this work we report on the structure and diversity of the ITS region in 87 representatives of the family Musaceae. We provide the first detailed information on ITS sequence diversity in the genus *Musa* and describe the presence of more than one type of ITS sequence within individual species. Both Sanger sequencing of amplified ITS regions and whole genome 454 sequencing lead to similar phylogenetic inferences. We show that it is necessary to identify putative pseudogenic ITS sequences, which may have negative effect on phylogenetic reconstruction at lower taxonomic levels. Phylogenetic reconstruction based on ITS sequence showed that the genus *Musa* is divided into two distinct clades – *Callimusa* and *Australimusa* and *Eumusa* and *Rhodochlamys*. Most of the intraspecific banana hybrids analyzed contain conserved parental ITS sequences, indicating incomplete concerted evolution of rDNA loci. Independent evolution of parental rDNA in hybrids enables determination of genomic constitution of hybrids using ITS. The observation of only one type of ITS sequence in some of the presumed interspecific hybrid clones warrants further study to confirm their hybrid origin and to unravel processes leading to evolution of their genomes.

## Introduction

Ribosomal ribonucleic acid (rRNA) is the central component of ribosomes - small intracellular particles, which convert the information carried in the genetic code into protein molecules [1]. In plant genomes, similar to other eukaryotes, the nuclear ribosomal RNA (rRNA) genes occur in thousands of copies and are organized in tandem arrays, typically clustered in two separate genomic loci. While the 5S rDNA locus encodes the 5S rRNA genes, the 45S rDNA locus contains genes for 18S, 5.8S and 26S rRNA, which are transcribed as a single unit and then spliced. The 18S, 5.8S and 26S rRNA genes are separated by internal transcribed spacers ITS1 and ITS2, and the 45S transcription units themselves are separated from each other by non-transcribed intergenic spacers (IGS), extending from the 3′ end of 26S rRNA to 5′ end of the 18S rRNA coding regions [2].

Although the ITS are not incorporated into the mature rRNA, they encode signals for proper processing of the rRNA transcripts, which itself depends on the secondary structure of ITS RNA [3]. In contrast to the ITS1 spacer, secondary structures of functional ITS2 spacer as well as 5.8S rDNA sequences are highly conserved within plants [4], [5]. ITS2 spacer forms a four helices structure with typical pyrimidine-pyrimidine bulge in helix II and the most conserved primary sequence includes the TGGT in the helix III [6]–[8]. The 5.8 S rDNA sequences contain three conserved motives in their nucleotide sequences that are essential for the correct folding of secondary structure [9], [10]. The conserved secondary structure of ITS2 and 5.8S sequences makes it possible to identify divergent ITS paralogs with non-functional pseudogenes [10]–[12].

The ribosomal genes arranged in tandem arrays are characterized by low intra-genomic diversity as a consequence of concerted evolution [13]–[15]. Individual copies of rRNA genes evolve more or less in unison and unequal crossing-over and high frequency of gene conversion are thought to be responsible for this [13]–[18]. The intraspecific and intra-population stability of rRNA locus on one hand and relatively fast evolution of ITS1 and ITS2, which are under lower selective pressure, made the ITS one of the most popular marker in phylogenetic studies [17], [19]–[21]. The analysis of the ITS was also employed to identify progenitors of hybrid species and to study the origin of polyploid species [e.g. 22]–[24].

However, the evolution of rRNA loci in hybrids and in allopolyploids in particular, may be complex and several evolutionary scenarios were observed which differ in the way the parental rRNA loci interact. In some species, such as *Arabidopsis*, *Brassica* or *Silene*, the divergent parental rDNA sequences remain conserved and evolve independently in hybrids without any interaction [25]–[27]. In other cases, such as in allopolyploids of *Nicotiana* or *Dendrochilum*, parental rDNA loci seem to recombine as judged from the occurrence of chimerical ITS sequences [28], [29]. The third evolutionary pathway is a dominance of one type of rDNA sequence that may lead to partial or even complete homogenization of rDNA locus of the second parent [18], [30]–[32]. The dominant rDNA sequence may represent one of the parental rDNA sequence when one rDNA type is lost or eliminated or may be a chimeric sequence resulting from intergenomic recombination [16]–[33]. Obviously, if the concerted evolution is not complete, different types of paralogous and orthologous rDNA sequences may be present in the genome, often including silenced and non-functional rDNA sequences referred to as pseudogenes. The presence of pseudogenes in the phylogenetic data set may result in altered phylogenetic inference [34].

Banana (*Musa* spp.) is an important cash crop and staple food for millions people living in the humid tropics. Most of edible banana cultivars are seed-sterile diploid, triploid and tetraploid intra- and inter-specific hybrids, which contain various combinations of the A and B genomes originating from seed bearing progenitors *M. acuminata* Colla and *M. balbisiana* Colla, respectively [35]. A few edible hybrids originated from crosses between *M. acuminata* and *M. schizocarpa* (S genome) and *M. acuminata* and presumably *M. textilis* (T genome) as well. The genus *Musa* belongs to the family Musaceae that includes three genera, *Musa* L., *Ensete* Horan and *Musella* C.Y. Wu ex H.W. Li, and has traditionally been divided into four sections based on morphology and basic chromosome number [36]: *Eumusa* (2n = 2x = 22), *Australimusa* (2n = 2x = 20), *Rhodochlamys* (2n = 2x = 22) and *Callimusa* (2n = 2x = 18, 20). However, the traditional classification of the genus *Musa* has been questioned based on DNA analyses [37]–[40]. Wong *et al.* [39] proposed to compound sections *Eumusa* and *Rhodochlamys* into one and sections *Australimusa* and *Callimusa* into second one. Comparable observations on the similarity of *Eumusa* and *Rhodochlamys* on one hand and *Australimusa* and *Callimusa* on other hand were made by others [41], [42]. Apart from this, the traditional taxonomy of *Musa* is also problematic for placement of newly described species [43], [44].

In addition to studies which employed AFLP, SSR and DArT markers [40], [45]–[47], only two studies on genetic diversity and phylogenetic relationships in *Musa* involved the analysis of rDNA region, namely the length and specific restriction pattern of the ITS1-5.8S-ITS2 region. Nwakanma *et al.*, [48] noted that a specific restriction pattern of the ITS region enabled differentiation between diploid AA and BB genomes as well as between the triploid AAA intraspecific hybrid clones and those that carry the B genome (AAB and ABB). More recently, the nucleotide information of the ITS region was used to study relationships of wild species of Musaceae [49], [50]. Nevertheless, there has been a lack of detailed knowledge on the ITS region in *Musa* and its potential for the analysis of genetic diversity and phylogenetic relationship remains unexplored.

In this work we set out to study in detail the rDNA ITS1-5.8S-ITS2 region in a total of 87 accessions of wild diploid species and triploid hybrid clones that represent all main taxonomic groups of the family Musaceae. We analyzed DNA sequences obtained either by classical dideoxy termination chain reaction or by massively parallel 454 sequencing with the aim to (1) thoroughly describe the nucleotide structure and diversity of the ITS region in *Musa*, *Ensete* and *Musella*; (2) compare the results obtained by different sequencing approaches; (3) analyze the presence of putative pseudogenic ITS sequence types and their influence on phylogenetic reconstructions; (4) assess the utility of ITS sequences for analysis of phylogenetic relationships within Musaceae and (5) use ITS to verify the genome constitution of inter- and intra-specific banana hybrids.

## Results

We have analyzed more than 2,500 nucleotide sequences from the ITS region of 87 accessions representing the family Musaceae, including 54 *Musa* diploids representing all sections of the *Musa* genus including four inter-subspecific hybrids, two *Ensete* and one *Musella* species (Table S1). In addition to the representatives of wild *Musa* species, ITS region of 30 hybrid banana clones was analyzed (Table S1). Three approaches were used to obtain ITS sequences. As the first step, the ITS region of all diploid genotypes was amplified using specific primers and four PCR products from individual PCR reactions of each accession were used for direct Sanger sequencing. Only 23 diploid genotypes produced readable ITS sequences with no polymorphism (Table S1), the remaining diploid accessions produced polymorphic ITS sequences. The ITS region of heterogeneous diploids and all hybrid clones was cloned and 15–85 DNA clones bearing ITS region were sequenced (Table S1). Finally, we have assessed the usefulness of massively parallel 454 sequencing for the analysis of the ITS loci.

### Length variation, sequence diversity and GC content of the ITS region

In general, the length of ITS1 and ITS2 spacer varied from 216 to 223 bp and from 205 to 227 bp, respectively. A total length of ITS1-5.8S-ITS2 sequence region ranged from 578 bp (*M. acuminata* ‘Truncata’, ITC 0393) to 601 bp (*M. mannii*, ITC 1411) in all accessions, except for five representatives of section *Australimusa*: *M. maclayi* (ITC 1207), *M. maclayi* type Hung Si (ITC 0614), *M. menei* (ITC 1021), *M. textilis* (ITC 1072) and *M. maclayi* F. Muell, where the ITS region was 544 bp long. This significant length difference specific for these *Australimusa* entries was due to a 41 bp deletion in ITS1.

One of the highest sequence diversities within diploid species was observed in *M. schizocarpa* (ITC 0856, ITC 0846) (Figure S1A, B/sequence similarity matrix). The heterogenous representatives of *Australimusa* and *Rhodochlamys* sections contained more diverse ITS sequence region than the representatives of *Eumusa*. Within the hybrids, banana clones ‘Cachaco’ (ITC 0643) and ‘Dole’ (ITC 0767), representatives of Bluggoe subgroup (ABB genomes), showed the highest level of sequence divergence, while the other three Bluggoe clones showed relatively low level of sequence heterogeneity (data not shown). Sequence divergence of the entire ITS region was higher among the hybrids with genome constitution AAA, AxB and AxT than among corresponding diploid genotypes (Figure S2). On the other hand, AxS banana hybrid clones showed lower sequence divergence than the S genomes, which contained putative pseudogenes (Figure S2) GC content of ITS1 varied from 55.35 to 67.70% and was slightly lower than the GC content of ITS2 (56.11 to 70.97%).

The 5.8S rDNA sequence region had a conserved length of 155 bp or 154 bp, and its GC content varied from 49.68 to 57.48% and was significantly lower than the GC content in ITS1 and ITS2. Among all Musaceae species studied, the lowest GC content of the ITS region was identified in accession representing the section *Australimusa* ().

### Secondary structure of ITS2 and 5.8S rDNA sequences in Musaceae and identification of pseudogenes

**Figure 1:**
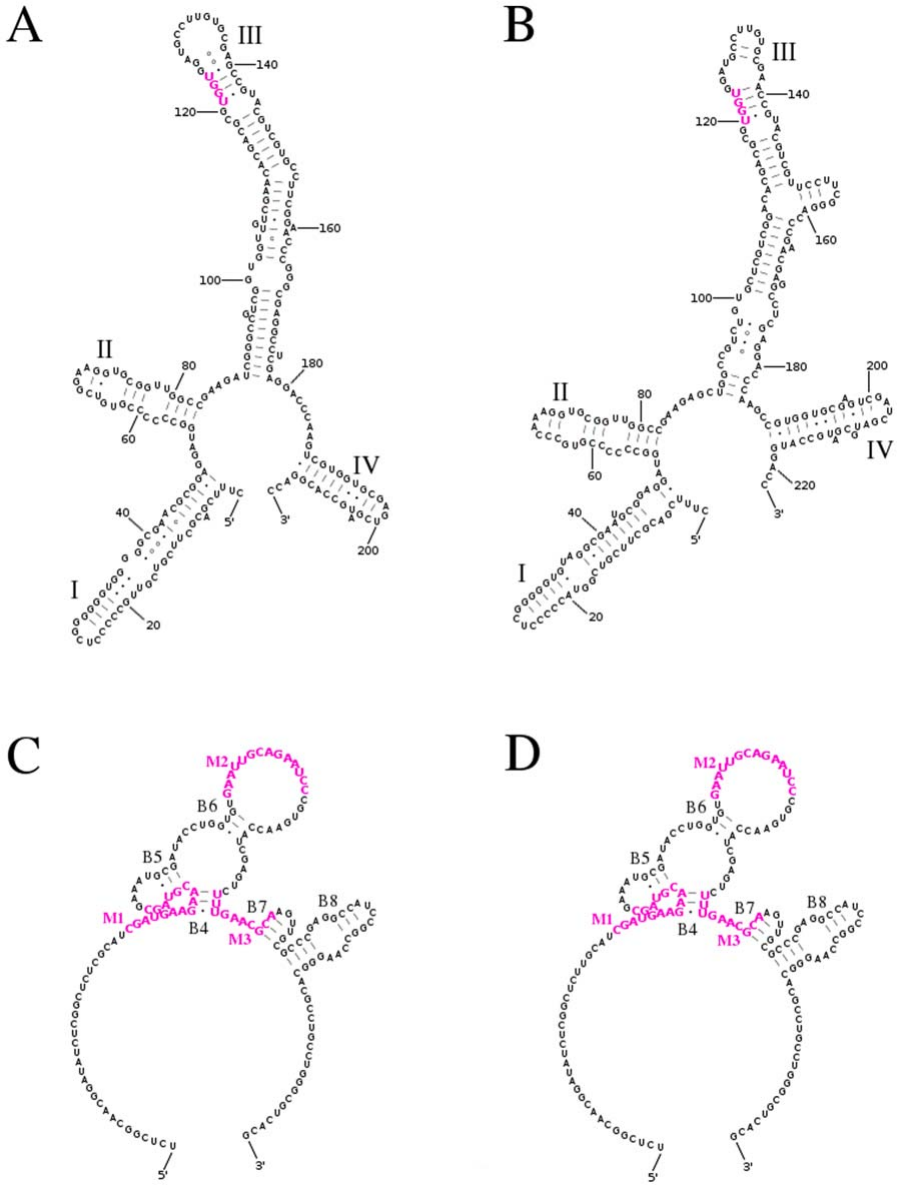
Examples of functional secondary structures of the ITS2 spacer and 5.8S rRNA gene. Positions within typical four-helices structures of ITS2 spacer of *M. acuminata* ‘Calcutta 4’ (A) and *E. gilletii* (B) are numbered every 20 nucleotides and helices are numbered I–IV. The common TGGT sequence motif in helix III is marked in red. The helices (B4–B8) in the conserved secondary structure of 5.8S rRNA gene sequences of *M. acuminata* ‘Calcutta 4’ (C) and *E. gilletii* (D) are numbered according to Wuyts *et al.* [82]. Conserved motives (M1–M3) are marked in violet.

The secondary structure of ITS2 and 5.8S rDNA sequence regions was reconstructed for all accessions. ITS2 sequences formed specific four-helices structure with typical pyrimidine-pyrimidine bulge in helix II and the most conserved primary sequence included the TGGT in the helix III (Figure 1A, B). Secondary structure of 5.8S rDNA sequence was reconstructed under specific settings for base pairing (Figure 1C, D). Moreover, 5.8S rDNA sequences were checked for the presence of three conserved motives [9]. The highly conserved sequence of 16 bp motif M1 (5′-CGATGAAGAACGTAGC-3′) is a part of two helices – helix B4 and B5 [9]. One type of ITS sequence in heterogenous diploids *M. acuminata* ‘Tuu Gia’ (ITC 0610) and ‘Pisang Mas’ (ITC 0563) had a “T” at position 11 and 16 in the motif M1. Similarly, one type of ITS in *M. acuminata* ‘Truncata’ (ITC 0393) and *M. schizocarpa* (ITC 0856 and ITC 0846) had an “A” at position 12. Motif M2 (5′-GAATTGCAGAATCC-3′), previously described by Jobes and Thien [5] is 14 bp long and located in the loop and 10 bp long motif M3 (5′-TTTGAACGCA-3′) is a part of the B4 and B7 helices [9] (Figure 1C, D). Some ITS types of *Musa* diploids had a changes at positions 9 and 14 in motif M2 and changes at positions 7 and 8 in the motif M3. The nucleotide changes in conserved motives of 5.8S rDNA of diploid and hybrid accessions are summarized in Table S2. The information on GC content, presence of conserved motives in the 5.8S rDNA sequence and ability of ITS2 and 5.8S rDNA sequence to fold into a conserved secondary structure allowed us to identify putative pseudogenes (Table S2).

### Comparison of the ITS1-5.8S-ITS2 sequence region obtained using different sequencing approaches

**Figure 2:**
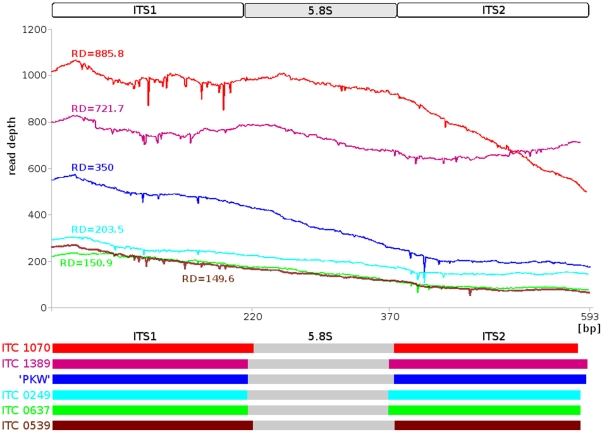
Reconstruction of the complete ITS1-5.8S-ITS2 region in six accessions of *Musaceae* after 454 sequencing. The graphs show the read depth (RD; number of 454 reads that were assembled over individual position) along the contig sequence. Color coding: *M. beccarii* ITC 1070 - red; *E. gilletii* ITC1389 - violet; *M. balbisiana* ‘Pisang Klutug Wulung’ - blue; *M. acuminata* ‘Calcutta4’ ITC 0249 - light blue; *M. ornata* ITC 0637 - light green and *M. textilis* ITC 0539 - brown.

In addition to Sanger sequencing, we used massively parallel sequencing (454) to study the diversity of the ITS region within six diploid Musaceae accessions (Table S1) and to compare the data obtained using the two different sequencing approaches. Reconstructed contigs of ITS regions from 454 data were characterized by high read depth (Figure 2). Analysis of the 454 data confirmed that the ITS region is highly conserved in all six accessions. Moreover, the 454 consensus ITS sequences were highly similar to those obtained using Sanger sequencing.

454 data of *M. acuminata* ‘Calcutta4’ resulted in one ITS sequence type that showed high similarity (99.67%) to the sequence obtained using direct Sanger sequencing - only two mismatches were found. ITS sequence region of *M. balbisiana* ‘Pisang Klutug Wulung’ obtained by 454 showed 99.83% similarity to the Sanger sequencing data, differing in one nucleotide insertion within the poly-G sequence region. The 454 sequencing of *M. ornata* and *E. ventricosum* resulted in two types of ITS sequence, showing 100% and 99.83% (single nucleotide substitution) similarity to the direct Sanger sequencing, output, respectively.

The highest number of 454 reads homologous to ITS region was obtained for *M. beccarii*, an observation which reflects higher copy number of 45S rRNA genes that are localized on three pairs of chromosomes in *M. beccarii* ITC 1070 [42]. The remaining species analyzed by 454 sequencing have only one pair of chromosomes bearing the 45S locus. 454 sequencing resulted in two types of ITS sequence in *M. beccarii*. The first type showed 99.65% similarity to the sequence obtained by direct Sanger sequencing (2 nucleotide changes). The second one contained 8 nucleotide substitutions showing lower similarity (98.63%) to the Sanger sequencing data and the presence of nucleotide substitutions at potential methylation sites, indicated its pseudogenic nature.

Out of the six Musaceae species evaluated using the two different sequencing approaches, only *M. textilis* gave polymorphic ITS sequence after direct Sanger sequencing. Subsequent cloning and Sanger sequencing of 15 clones carrying the ITS region resulted in two different consensus ITS sequences (0539con1 and 0539con2) (Table S1). This observation was confirmed by 454 sequencing, which resulted in two types of ITS sequence differing in three nucleotide substitutions. Both 454-ITS types showed high similarity (99.49% and 99.67%) to 0539con1 ITS sequence. Second type of ITS sequence obtained after Sanger sequencing (putative pseudogene; Table S2) was not found in the 454 dataset despite the relatively high read depth of the ITS-454 data (RD = 149.6; Figure 2).

### Phylogenetic reconstruction

#### Position of the Musaceae clades with respect to closely related Zingiberales

**Figure 3:**
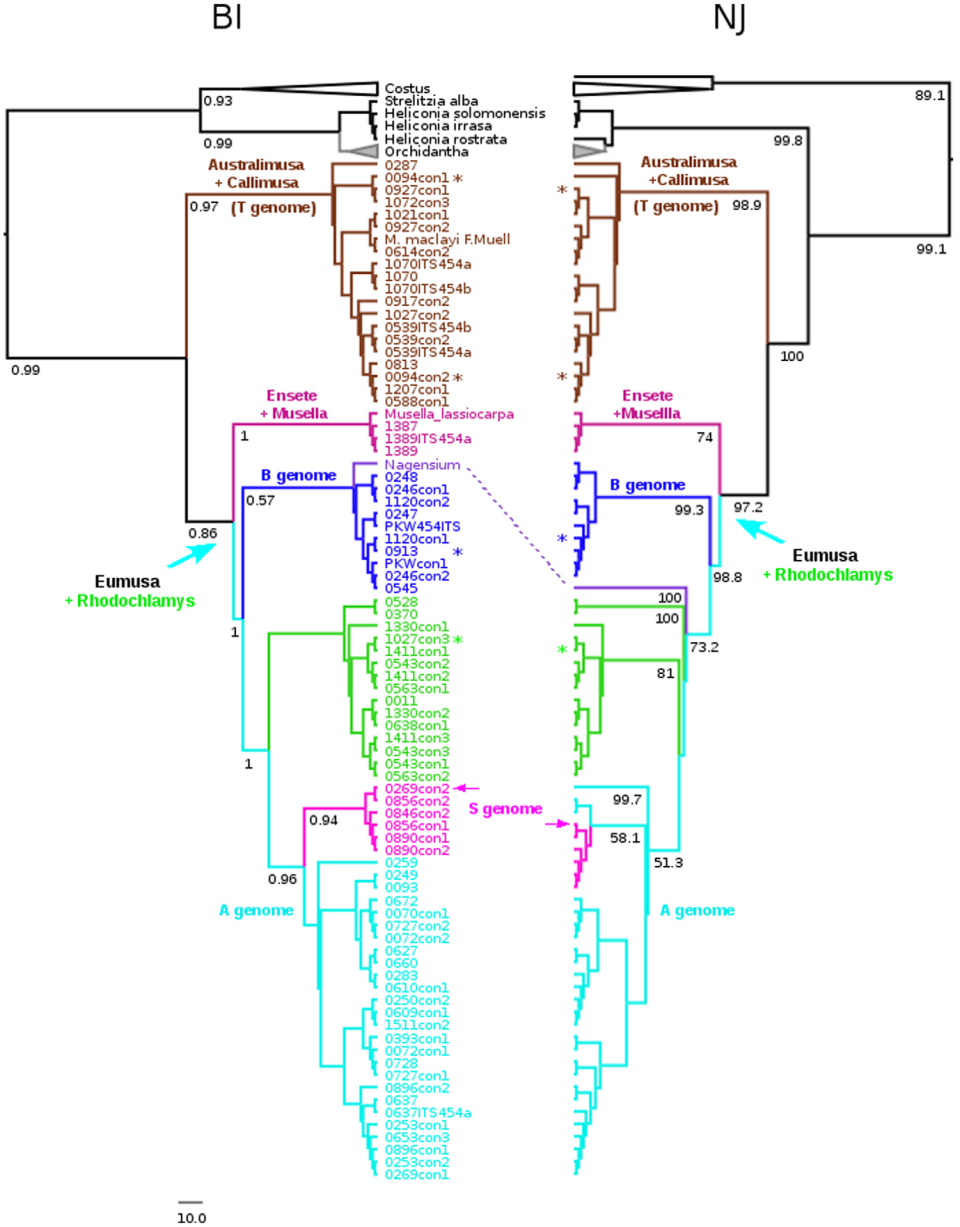
Phylogenetic analysis of diploid representatives of Musaceae and selected species of closely related families Strelitziaceae, Lowiaceae, Heliconiaceae and Costaceae based on the ITS1-ITS2 sequence region. Neighbor-Joining (NJ) tree constructed from a Jukes-Cantor distance matrix of the concatenated region containing ITS1 and ITS2 spacer sequence. Bayesian inference (BI) analysis of the same data set was performed in BEAST v1.5.3 using GTR+I+G model of nucleotide substitution. Both trees are rooted at the midpoint and values below the branches indicate the bootstrap support of NJ analysis (A) and posterior probability of BI (B), respectively. Putative pseudogenes were not included in this analysis. Main clades and subclades were labeled by different colors that correspond to Figure 2. *Ensete*/*Musella* clade - violet; *Australimusa*/*Callimusa* - brown; *Rhodochlamys* - light green; B genomes - blue; A genomes - light blue and S genomes - pink.

We have used two different methods to reconstruct phylogenetic relationships within Musaceae considering other closely related families of the order Zingiberales - Neighbor-Joining (NJ) and Bayesian inference (BI). The best-fit model of nucleotide substitution selected by jModeltest and implemented in Bayesian analysis was GTR+I+G. If putative pseudogenic ITS types were excluded from data set, NJ and BI resulted in fully resolved trees with high NJ bootstrap support and Bayesian posterior probabilities for all main clades (Figure 3). In these analyses, ITS1-ITS2 concatenated region of all diploid Musaceae accessions, including the four inter-subspecific diploid hybrids and closely related families from Zingiberales (dataset 1) was used for NJ constructed on Jukes-Cantor distance matrix and BI analysis performed in BEAST.

The reconstructed phylogeny confirmed the monophyly of Musaceae and revealed three main clades within the family. The monophyly of *Ensete* and *Musella* was not supported as they shared the same clade. Species from the section *Australimusa* shared the same clade with the species from section *Callimusa* as did the species from sections *Eumusa* and *Rhodochlamys* (Figure 3). Moreover, the positions of the clade bearing *Ensete* and *Musella* species and that of *Australimusa* and *Callimusa* species was reverse as compared to the work of Liu *et al.* [50]. Based on sequence analysis of 19 intron containing nuclear genes Christelová *et al.* [51] created a phylogenetic tree in which the position of the clade bearing *Musella* and *Ensete* genera was sister to the *Musa* genus and the *Australimusa*/*Callimusa* sections formed a separate subcluster within the *Musa* genus clade.

In case of *Australimusa*/*Callimusa* clade, *M. coccinea* appeared as a distinct species within the clade. All accessions of cultivated Fe'i banana, except ‘Utafan’ (ITC 0913) clustered with the *Australimusa* species and both, BI and NJ phylogenetic analysis supported their close relationships to *M. textilis*. Moreover, the *Australimusa*/*Callimusa* clade contained one accession from the *Eumusa* section - *M. balbisiana* 10852 (ITC 0094) (Figure 3). Within the *Eumusa*/*Rhodochlamys* clade, representatives of the B genome formed strongly supported subclade separated from other *Eumusa* and *Rhodochlamys* species. However, the B genome subclade contained also one representative of Fe'i bananas - ‘Utafan’ (ITC 0913). Another well separated subclade was formed by representatives of section *Rhodochlamys*. However, this strongly supported subclade contained one accession of section *Australimusa* – *M. textilis* ‘Née’ (ITC 0563), and one representative of Fe'i bananas – ‘Asupina’ (ITC 1027). Moreover, two *Rhodochlamys* accessions - *M. ornata* (ITC 0637) and *M. velutina* (ITC 0627) clustered with the A genome representatives, which supports close relationships of *Eumusa* and *Rhodochlamys* species. Within the representatives of *M. acuminata*, both phylogenetic analyses supported the close relationship of *burmannica*, *burmannicoides*, *siamea* and *malaccensis* subspecies.

The BI analysis revealed a well separated and strongly supported subclade of the S genome representatives (*M. schizocarpa*) together with *M. acuminata* ‘Niyarma yik’ (ITC 0269). In the NJ analysis, representatives of the S genome clustered not only with *M. acuminata* ‘Niyarma yik’ (ITC 0269), but also with two other representatives of A genome - *M. acuminata* ‘Malaccensis’ (ITC 0250) and *M. acuminata* ‘Galeo’ (ITC 0259). BI and NJ trees differed also in the position of *M. nagensium*. In the BI tree, *M. nagensium* shared the same clade with the B genome representatives, supporting their close relationship. On the contrary, *M. nagensium* formed a highly supported separate subclade in the NJ tree, indicating its phylogenetic isolation from other *Eumusa* species (Figure 3). Finally, the analysis revealed that presence of putative pseudogenic ITS data had no significant effect on positions of main clades in the BI and NJ phylogenetic trees. Nevertheless, the positions of some accessions were changed within the main clades, especially within the A genome representatives (Figure S3).

#### Evolutionary relationships of banana hybrid clones

Thanks to the specific mode of the rDNA loci evolution, the ITS data can be used to analyze the genome composition and evolutionary relationships of hybrid plant species [22]–[24], [26], [27]. Here we present the first sequence data from the ITS region and its utilization for analysis of genome composition and evolutionary relationships in different banana hybrid clones. Both phylogenetic trees (BI and NJ) based on the ITS1-ITS2 concatenated region of dataset 2 (see Materials and Methods) contained cluster of ITS sequences corresponding to B genome well separated from the other genomes of *Eumusa* section. For example, banana clone ‘Saba’ ITC 1138, traditionally classified as ABB hybrid, contained three types of ITS sequence. One of them (1138con1) clustered with the ITS sequences corresponding to B genome and two other ITS types of ‘Saba’ (1138con2 and 1138con3) clustered with the A genome ITS types (Figure S4, Table S3). Similarly, *M. jackeyi* ITC 0851 which has been classified as AT hybrid contained three different types of ITS sequence region. One ITS type clustered together with the ITS corresponding to T genome (*Australimusa*/*Callimusa* clade) as well as the ITS sequence type (0851con3) identified as putative pseudogene. The third ITS sequence type of *M. jackeyi* (0851con2) clustered with the ITS types corresponding to A genome (Figure S4, Table S3). Similarly to dataset 1, most of the *Rhodochlamys* species formed a separated subclade. The cluster containing diploid representatives of the S genome and AS hybrids also contained ITS types from banana clones that were not classified as A×S hybrids (Figure S4). This supports close relationships of *M. acuminata* and *M. schizocarpa*. In comparison to the phylogenetic analysis using dataset 1, the position of the main clades within Musaceae in dataset 2 remained conserved in BI tree. Phylogenetic analysis using NJ of dataset 2 showed reversed position of *Ensete*/*Musella* and *Australimusa*/*Callimusa* clades (Figure S4).

BI and NJ phylogenetic study revealed that most of the banana hybrid clones contained conserved parental ITS sequence types. This information could facilitate rapid determination of genome composition of newly created hybrid clones. However, not all of the evaluated hybrid clones carried conserved parental ITS types (Figure S4, Table S3). The hybrid clones with the reported AAB genome constitution (‘Maritú’ ITC 0639, subgroup Iholena and ‘3 Hands Planty’ ITC 1132, subgroup Plantain) and ABB (‘Cachaco’ ITC 0643, subgroup Bluggoe) did not contain ITS sequence corresponding to the B genome. These results suggest partial homogenization of the rDNA locus in the clone ‘Maritú’ which contained two different ITS sequence types where one of them has putative pseudogenic character. On the other hand the hybrid clones ‘3 Hands Planty’ ITC 1132 (subgroup Plantain) and ‘Cachaco’ ITC 0643 (subgroup Bluggoe) contained four and six ITS sequence types, respectively. The presence of chimeric ITS sequences with higher number of putative pseudogenic sequences in these cases can be due to recombination as described in other plant hybrids [28], [29]. In the Bluggoe subgroup, two other hybrid clones - ‘Cachaco Enano’ ITC 0632 and ‘Kivuvu’ ITC 0157 exhibited partial concerted evolution when the ITS sequence of A genome was not found in their genomes. Banana clone ‘Butuhan’ ITC 1074, reported as BB×TT hybrid contained only ITS sequence corresponding to B genome. Similarly, clone ‘Tonton Kepa’ ITC 0822 reported as AS hybrid contained only S genome ITS sequence (Figure S4, Table S3).

## Discussion

The ITS1-5.8S-ITS2 sequence region is one of the most popular loci used in molecular phylogenetic studies [17]. Despite this, the ITS region has not been analyzed in detail in the genus *Musa* and has only been used once to examine its phylogenesis [50]. However, the study suffered from the experimental approach as it was based solely on direct sequencing of the ITS locus, and the authors did not consider sequence homogeneity/heterogeneity of the region. As the potential of the ITS analysis in the taxonomy of Musaceae remains unexplored, we chose to study the structure and diversity of the ITS1-5.8S-ITS2 region in wild species of *Musa* as well as in cultivated banana clones. We evaluated different sequencing approaches including the next generation sequencing technology and their impact on the phylogenetic reconstruction.

### ITS length variation, secondary structure and identification of pseudogenes

Recent studies revealed that divergent ITS paralogs may contain non-functional pseudogenes [10]–[12] whose presence in phylogenetic data sets can result in altered phylogenetic inferences [34]. ITS pseudogenes were identified experimentally using transcript analysis [17]. Alternatively, *in silico* methods such as the analysis of nucleotide divergence, insertions and deletions (length of the ITS sequence region), analysis of methylation-induced substitution pattern, folding of conserved secondary structures and sequence free energy were used [52]. Due to the lack of transcript data, we evaluated ITS length, GC content and secondary structure of ITS2 and 5.8S rDNA regions. The total length of ITS regions as well as the length of putative functional ITS1 and ITS2 spacers agreed with the observations in other angiosperms [14]. However, we have identified polymorphic ITS regions in most of the *Musa* accessions and it is possible that at least some of the polymorphisms were due to the occurrence of pseudogenic sequences. In agreement with other studies [17], the putative pseudogenic ITS sequences varied in length of ITS1 spacer, had lower GC content, did not contain conserved motives in the 5.8S rDNA sequence and/or lost the ability to fold into functional ITS2 and/or 5.8S rDNA secondary structures.

### Intra-individual polymorphism and evolution of ITS

Despite the concerted evolution of rDNA, ITS polymorphism within individuals is quite common [14], [15], [52], [53] and may be due to various mechanisms. A single diploid genome contains divergent paralogs when the speciation is faster than concerted evolution or when paralogous rDNA sequences are present in non-homologous loci in the heterozygous species. Our results suggest incomplete concerted evolution in some wild diploid banana species, in which we revealed increased levels of ITS polymorphism and divergent paralogs of ITS sequences. The latter might be formed as a consequence of inter- and/or intra-genomic duplication events. Alternatively, the polymorphic character of ITS region in some wild diploids could be due to inter-subspecific hybrid origin and asexual reproduction, which bypasses meiotic recombination.

Owing to unclear origin of hybrid banana varieties and their clonal character, it is difficult to reconstruct the evolution of the 45S rDNA locus in *Musa*. Our results suggest that all three modes of evolution were involved in polyploid hybrid clones. Most of the interspecific banana hybrids presumably originating from crosses *M. acuminata*×*M. balbisiana*, *M. acuminata*×*M. schizocarpa* and *M. acuminata*×*M. textilis* contained conserved ITS sequences of both parents, indicating incomplete concerted evolution of rDNA loci. No or limited homogenization of ITS sequence was described for allopolyploid genomes of European dogroses, where most of the genomes are excluded from meiotic recombination [54]. The independent evolution of parental rDNA sequences in banana hybrid clones, which is probably due to the absence of sexual reproduction, could facilitate determination of genomic constitution of new hybrid banana cultivars. However, the fact that presence of chimeric ITS sequences and/or partial concerted evolution was observed in some hybrids may limit the potential of this approach. These hybrids show various degree of residual fertility and it is possible that their evolution involved episodes of sexual reproduction as suggested by the backcross hypothesis [55]. Our results indicate a complete concerted evolution in two banana clones: ‘Butuhan’ (BB×TT) and ‘Tonton Kepa’ (AA×SS) in which only ITS sequence of B genome and S genome was present, respectively. However, this observation may be explained either by a more complicated evolution, which included backcrossing to one of the parental species [55], or a non-hybrid origin. Clearly, the genomic constitution of both clones needs to be confirmed using additional molecular analysis.

### Comparison of ITS region obtained using different sequencing approaches

Plant species and hybrid and polyploid ones in particular, often contain several rDNA loci with multiple divergent ITS sequence types. Multiple ITS sequence types may originate by different evolutionary mechanisms and their utilization in molecular phylogeny may shed light on polyploid ancestry, genome relationships, genomic constitution and evolution of hybrid species [30]–[32], [52], [56].

The ITS sequence complexity (diversity) may lead to errors in phylogenetic interference due to PCR bias and/or PCR drift [reviewed in 17]. To check the effect of ITS heterogeneity, we compared ITS sequence data obtained by classical approach (sequencing PCR products and/or PCR/cloning strategy) with the data obtained by massively parallel 454 sequencing [57]. We obtained whole genome 454 data from five *Musa* and one *Ensete* species with the length of sequencing reads sufficient to assembly individual ITS sequence types without PCR/cloning strategy. The ITS sequence data obtained using classical approach were highly similar to those obtained using 454 and resulted in the same phylogeny. However, the 454 data obtained in *M. beccarii* (ITC 1070) indicated the presence of two ITS sequence types while direct Sanger sequencing resulted in one readable ITS sequence with no polymorphism. This could be a consequence of PCR bias, when a single repeat type is preferentially amplified. Other exception was *M. textilis* (ITC 0539), in which one sequence type of ITS obtained after Sanger sequencing was not found in the 454 dataset despite the relatively high read depth. This outcome suggests some modification of the ITS sequence during the classical PCR/cloning methodology and indicate that massively parallel sequencing may be a preferable approach to study 45S rDNA sequence region as well as the other complex gene families.

### Phylogenetic reconstruction using ITS

In order to analyze the position of the family Musaceae and its main clades and subclades within the order Zingiberales, we studied the ITS sequences in other families of Zingiberales (Strelitziaceae, Lowiaceae, Heliconiaceae and Costaceae). In line with the results of Liu *et al.* [50], who studied ITS and chloroplast loci, our analyses showed monophyletic origin of Musaceae. Both BI and NJ phylogenetic trees showed close relationships of species belonging to sections *Australimusa* and *Callimusa* and sections *Eumusa* and *Rhodochlamys*, thus confirming previous findings [39], [41], [42], [49], [50]. At the same time, both trees showed reverse positions of the *Ensete*/*Musella* clade and *Australimusa*/*Callimusa* clade, probably due to the use of only one genomic locus (ITS) in the present work. Among the *Eumusa*/*Rhodochlamys* species, we revealed a distinct position of *M. balbisiana*, which has traditionally been placed within the *Eumusa*, and most of *Rhodochlamys* species. This suggest earlier separation of the representatives of B genome from the A genomes and *Rhodochlamys* representatives in the evolution of Musaceae and is on line with the divergence time estimates of Christelová et al. [51]. The placement of *M. acuminata* and *M. balbisiana* into two distinct subclades within *Eumusa*/*Rhodochlamys* clade was suggested by Li *et al.* [49] and close relationship of *M. acuminata* (section *Eumusa*) with *M. laterita* and *M. ornata* (both section *Rhodochlamys*), was also observed earlier [39], [40], [58]. Separation of *Eumusa* and *Rhodochlamys* sections was questioned already by Cheesman [36] and Shepherd [59] and the present as well as earlier results obtained with molecular tools indicate close relationships of the representatives from both sections [39], [40], [49], [50].

In our study, three accessions classified as representatives of section *Australimusa* (*M. textilis* ‘Née’ ITC 0563 and Fe'i bananana clones ‘Utafan’ ITC 0913 and ‘Asupina’ ITC 1027) were found clustering with B genomes subclade or *Rhodochlamy*s subclade. Similar situation was observed for *M. balbisiana* 10852 (ITC 0094) which grouped with *Australimusa* (see Results and Figure 3). This unexpected clustering could be due to mislabeling during their collection and/or during *in vitro* propagation in the gene bank. Alternatively, these accessions could be interspecific hybrids and/or their backcross progenies [55].

Despite a range of studies [39], [40], [49], [50], taxonomic and phylogenetic positions of some taxa within the Musaceae are still unclear and/or controversial. One of them is *Musella*, which has been classified based on morphology as a separate genus of Musaceae [60]. The present study indicates close relationship of *Musella* and genus *Ensete* and does not confirm the monophyly of *Musella*. The same observation was made by molecular phylogenetic analysis based on the ITS and clDNA sequences [49], [50] as well as work based on genic sequences [51].

The problematic phylogenetic position was also reported for the two representatives of section *Callimusa*, which differ by basic chromosome number - *M. beccarii* (2n = 2x = 18) and *M. coccinea* (2n = 2x = 20) [42]. Our study supports close relationship of *Callimusa* with the section *Australimusa* (2n = 2x = 20), where *M. coccinea* is relatively distinct from other accessions. The distinctiveness of *M. coccinea* is supported by morphological characters [36], AFLP analysis [39] and by the ITS and clDNA analysis of Li *et al.* [49]. In contrast to Liu *et al.* [50], our data do not support close relationship of *M. beccarii* and *M. maclayi* and indicate close relationships of cultivated Pacific Fe'i bananas to *M. textilis* and *M. maclayi*, while not confirming genetic vicinity of *M. textilis* and *M. balbisiana*.

An important outcome of this study is the identification of putative pseudogenic ITS paralogs in some banana diploid representatives and most of banana hybrid clones. Pseudogenes are expected to evolve independently at different rates than their functional counterparts and can accumulate mutations. This may lead to random clustering across phylogenetic trees and result in incorrect or incongruent phylogenies [34], [61], [62]. However, Ochieng *et al.* [11] found that the ITS pseudogenes can recover more corroborated phylogeny of closely related species when functional paralogs suffer selective constraints and provide too low variation. In this work we demonstrate that the presence of putative pseudogenic ITS sequences has no significant effect on the structure of main clades in the phylogenetic analysis of Musaceae. On the other hand, their presence may change the position of subclades (Figure S3). These results show that pseudogenic ITS sequences should be used with caution when analyzing phylogenesis in *Musa*. As both Liu *et al.* and Li *et al.* [49], [50] used only direct Sanger sequencing, differences in the placement of some diploid species in their phylogenetic trees as compared to our results could be due to the inability to detect different ITS sequences types.

### Conclusions

The present study provides the first insights into the nucleotide structure and diversity of the ITS1-5.8S-ITS2 region in Musaceae and reveal the presence of more than one type of the ITS sequence within some *Musa* species and hybrid banana clones. We show that it is necessary to use clone-based sequencing strategy for most of the species within Musaceae and identify putative pseudogenic ITS sequences that may have negative effect on phylogenetic reconstruction. We show that both Sanger and 454 sequencing lead to similar phylogenetic inferences. Phylogenetic analysis based on the ITS sequences does not support *Musella* as a distinct genus within the Musaceae and confirms close phylogenetic relationship of the species from sections *Australimusa* and *Callimusa*, and *Eumusa* and *Rhodochlamys*, where the B genome representatives form a distinct subclade. The reversed positions of the *Ensete*/*Musella* clade and *Australimusa*/*Callimusa* clade in the phylogenetic trees suggest that phylogenetic reconstruction based on a single genomic locus (ITS) may lead to inconsistent phylogenetic inference. On the other hand, the use of ITS locus in phylogenetics has proven powerful in reconstructing relationships on lower taxonomic levels (groups and subgroups). Most of the intraspecific banana hybrids analyzed in this work contain conserved parental ITS sequences, indicating incomplete concerted evolution of rDNA loci. Independent evolution of parental rDNA loci in hybrids enables verification of genomic constitution of hybrids using ITS. The observation of only one type of ITS sequence in some of the presumed interspecific hybrid clones warrants further study to confirm their hybrid origin and evolution of their genomes.

## Materials and Methods

### Plant material

*In vitro* rooted plants of most of the *Musa* and *Ensete* species used in the study as well as the hybrid clones (Table S1) were obtained from the International Transit Centre (ITC, Katholieke Universitiet, Leuven, Belgium). *M. balbisiana* ‘Pisang Klutug Wulung’ was obtained from Dr. François Côte (CIRAD, Guadeloupe) as rooted plants. Seeds of *M. nagensium* and *M. maclayi* F. Muell were obtained from Prof. Markku Häkkinen (Botanic Garden, University of Helsinki, Finland) and plants of *Musella lasiocarpa* were purchased from a commercial nursery. Plants and/or seeds were transferred to soil and grown in a greenhouse.

### Amplification, cloning and Sanger sequencing of ITS1-5.8S-ITS2 region

Genomic DNA of all *Musa*, *Ensete* and *Musella* species used for the ITS analysis was isolated from fresh cigar leaves using the Invisorb Spin Plant Mini Kit (Invitek, Berlin, Germany) following the manufacture's recommendations. The ITS region was amplified from the genomic DNA using PCR with specific primers ITS-L and ITS-4 [48]. PCR reaction mix consisted of 10 µg genomic DNA, 1.5 mM MgCl_2_, 0.2 mM dNTPs, 1 µM primers ITS-L and ITS-4, 1× PCR buffer and 2 U/100 of Dynazyme™ II DNA polymerase (Finnzymes, Espoo, Finland). Amplification was performed using PTC-200 thermal cycler (BIO-RAD, Hercules, USA), with the following conditions: 94°C for 5 min (1 cycle), 94°C for 50 s, 52°C for 50 s, 72°C for 50 s (35 cycles) and 72°C for 10 min (1 cycle), and PCR products were resolved in 1.5% agarose gels.

In the first experiment, PCR products of all diploid accessions were purified using ExoSAP-IT® (USB, Cleveland, USA) according to the manufacturer's instructions and used for direct sequencing. PCR products containing polymorphic ITS sequences were cloned into TOPO vector and transformed into *E. coli* electrocompetent cells (Invitrogen Life Technologies, Carlsbad, USA). For each heterogenous accession, 15 to 85 cloned PCR products were sequenced. Sequencing was carried out using the BigDye Terminator v3.1 Cycle Sequencing kit (Applied Biosystems, Foster City, USA) according to the manufacturer's instructions and run on ABI 3730xl DNA analyzer (Applied Biosystems). Nucleotide sequences were edited using Staden Package [63].

### 454 sequencing and data analysis

Genomic DNA of *M. acuminata* ‘Calcutta4’ (ITC 0249), *M. balbisiana* ‘Pisang Klutug Wulung’, *M. ornata* (ITC 0637), *M. textilis* (ITC 0539) and *M. beccarii* (ITC 1070) was prepared from nuclei isolated from healthy young leaf tissues according to Zhang *et al.* [64]. Intact nuclei of *E. gilletii* (ITC 1389) were isolated by flow cytometric sorting following the protocol of Šafář *et al.* [65]. Isolated nuclei were incubated with 40 mM EDTA, 0.2% SDS and 0.25 µg/µl proteinase K for 5 hrs at 37°C, and DNA was purified by phenol/chloroform precipitation.

454 shotgun sequencing libraries were prepared by the GS Titanium library preparation kit (Roche Diagnostics, Rotkreutz, Switzerland). Single-stranded libraries were quantified by a qPCR assay [66] and processed utilizing the GS Titanium SV/LV emPCR and XLR70 sequencing kits according to the manufacturer's instructions (Roche Diagnostics). Sequencing was performed on a half 70×75 picotiter plate for each *Musa* and *Ensete* species. Sequencing reads with the similarity to ITS1-5.8S-ITS2 region and 45S rRNA genes, respectively were identified using RepeatMasker [67] and the consensus sequences of the ITS1-5.8S-ITS2 region were assembled using cap3 program [68].

The read depth (RD) of the ITS sequence region in all six accessions was estimated according to Macas *et al.* [69] using home-made perl script and plots showing RD along the ITS locus (Figure 2) were created using the CALC program (http://www.openoffice.org/product/calc.html).

### ITS sequence analysis and secondary structure reconstruction

Nucleotide sequences obtained from both sequencing approaches as well as the nucleotide sequences of ITS regions of other known species from the order Zingiberales that were downloaded from the GenBank database (Table S4), were edited using Staden Package [63] and the sequence boundaries of the spacers were determined by comparison to known rice ITS sequence (GB code: AF169230). Multiple sequence alignments of the ITS regions were done using MUSCLE v3.70+fix1-2 [70] and graphically shown in SeaView v4.2.1 [71]. Multiple sequence alignment was used in accessions with polymorphic ITS sequences to identify individual ITS types, which were then used for further analysis. Nucleotide diversity (π) of ITS region was estimated in MEGA 4 [72].

GC content and sequence identity of ITS regions within heterogenous accessions was calculated using BioEdit (http://www.mbio.ncsu.edu/BioEdit/bioedit.html). Secondary structures of ITS2 region were predicted using mFOLD program version 3.2 [73], [74] at the default temperature (37°C) as well as at the 25°C and XRNA program version 1.2.0 Beta (http://rna.ucsc.edu/rnacenter/xrna/xrna_download.html). No differences in folding at both temperatures were observed. The 5.8S rDNA sequences were checked for the presence of the three conserved angiosperm motifs according to Harpke and Peterson [9]. The secondary structure of 5.8S rRNA gene was reconstructed under specific settings for base pairing in XRNA program: for helix B4, F 38 98 3; helix B5, F 41 54 3; helix B6, F 62 89 3; helix B7, F 103 111 3; and for helix B8, F 112 135 4 and F 119 128 3.

Consensus sequences of the ITS regions have been deposited in the GenBank (accession numbers: FR727838–FR727974) as well as on our web site (http://olomouc.ueb.cas.cz/phylogeny-musaceae) where they are freely available.

### Phylogenetic analysis

Two different datasets were generated: in order to analyze the position of the clades within Musaceae with respect to other phylogenetic groups of Zingiberales, dataset 1 comprised ITS1-ITS2 concatenated regions of all diploid Musaceae accessions as well as the known ITS1-ITS2 sequences of closely related families Strelitziaceae, Lowiaceae, Heliconiaceae and Costaceae [75], [76] (Table S4). Dataset 2 comprised ITS1-ITS2 sequences of all Musaceae accessions including hybrids and was used to analyze the genome composition and evolutionary relationships of hybrid banana species. All datasets were analyzed using Neighbour joining (NJ) and Bayesian inference (BI). Neighbour joining (NJ) method was carried out using SplitsTree4 v4.1.11 [77] based on the Jukes-Cantor and uncorrected *p*-distances. Non-parametric bootstrapping with 1000 pseudoreplicates was performed to assess the nodal support.

The most appropriate model of nucleotide substitution for each dataset was determined using jModelTest v0.1.1 [78]. Likelihood calculations were carried out using integrated PhyML [79] for 11 substitution schemes (88 different models) and the model selected under Akaike information criterion (AIC) [80] was implemented in the BI settings. Bayesian inference analysis was performed in BEAST v1.5.3 [81] with four independent Markov Chain Monte Carlo (MCMC) runs, starting from randomly chosen topologies. The MCMC were run for 10,000,000 generations, data were sampled every 1,000 generations. Log-file outputs were inspected in Tracer [81] to confirm the correct convergence of the analysis. Treefiles from individual MCMC runs were subsequently combined by LogCombiner [81]. The first 25% of the generations were discarded as the burn-in, the maximum clade credibility tree and corresponding posterior probabilities were calculated using TreeAnnotator [81]. Phylogenetic trees were drawn and edited using FigTree (http://tree.bio.ed.ac.uk/software/figtree/) program.

Sequencing data analysis and statistical analysis described above were performed on LINUX systems.

## Supporting Information

Figure S1 **An example of similarity matrix of heterogenous ITS sequences of** ***M. jackeyi*** **(A) and** ***M. schizocarpa*** **(B).**
(TIFF)

Figure S2 **A box plot showing sequence diversities (π) within** ***Musa*** **representatives with different genome constitution and hybrid clones.** The inter-subspecific diploids were not included in the analysis. Three diploid species which gave unexpected clustering in the phylogenetic tree (see Results and Discussion) were included in the data set according the results of NJ and BI analysis. * Only three representatives of S genome were included in our study. Two of them contained 3 ITS types including putative pseudogenic sequences and show the highest nucleotide difference among the analyzed species.
(TIFF)

Figure S3 **A BI and NJ phylogenetic trees of all ITS types including putative pseudogenes, obtained in diploid Musaceae accessions and selected species of closely related families Strelitziaceae, Lowiaceae, Heliconiaceae and Costaceae based on the ITS1-ITS2 sequence region.** NJ tree was constructed from a Jukes-Cantor distance matrix and BI analysis of the same data set was performed in BEAST v1.5.3 using GTR+I+G model of nucleotide substitution. Values below the branches indicate the bootstrap support of NJ analysis and posterior probability of BI, respectively. Main clades and subclades are labeled by different colors as used in Figure 3 and putative pseudogenic types of ITS sequences are marked by asterisks.
(TIFF)

Figure S4 **A BI and NJ phylogenetic analysis trees of all evaluated Musaceae accessions including banana hybrids and selected species of the closely related families Strelitziaceae, Lowiaceae, Heliconiaceae and Costaceae based on the ITS1-ITS2 sequence region.** NJ tree was constructed from a Jukes-Cantor distance matrix and BI analysis of the same data set was performed in BEAST v1.5.3 using GTR+I+G model of nucleotide substitution. Values below the branches indicate the bootstrap support of NJ analysis and posterior probability of BI, respectively. Main clades and subclades are labeled by different colors as used in Figure 3 and putative pseudogenic types of ITS sequences of hybrids are marked by asterisks.
(TIFF)

Table S1 **Diploid representatives of the family Musaceae and** ***Musa*** **hybrid clones used in the study.** Diploid representatives that are probably inter-subspecific hybrids [83] are marked by asterisk.
(DOC)

Table S2 **Sequence characteristics of ITS1-5.8S-ITS2 regions in Musaceae.**
(DOC)

Table S3 **Table showing genome composition of hybrid clones analyzed in this study and corresponding ITS sequence types identified after NJ and BI analysis.**
(DOC)

Table S4 **Representatives of the order Zingiberales.**
(DOC)
