# Supplementary figures and images for: The ITS1-5.8S-ITS2 Sequence Region in the Musaceae: Structure, Diversity and Use in Molecular Phylogeny
Source: PLoS One. 2011 Mar 22;6(3):e17863. doi: 10.1371/journal.pone.0017863 (PMC3062550; doi:10.1371/journal.pone.0017863)

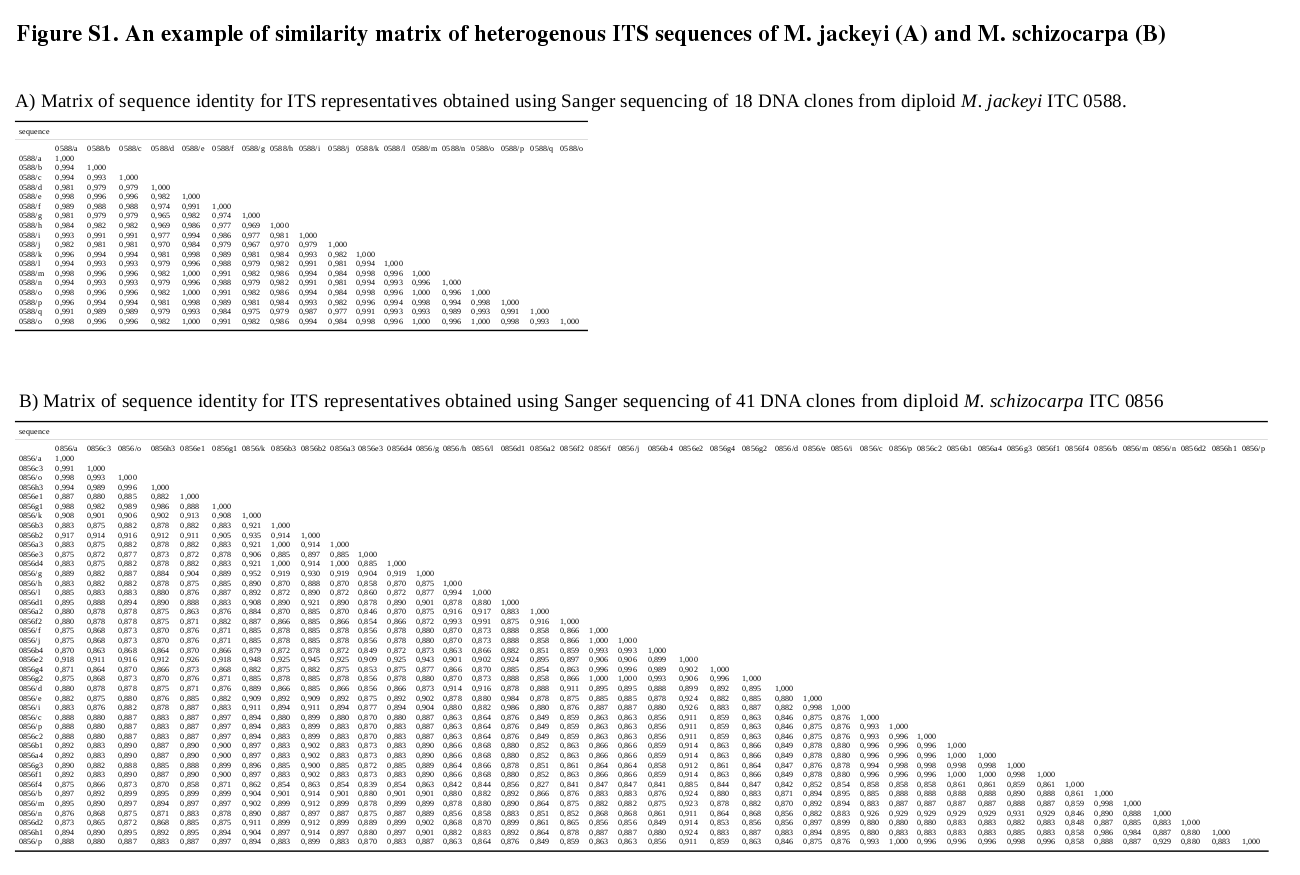

Supplement: Figure S1 — An example of similarity matrix of heterogenous ITS sequences of M. jackeyi (A) and M. schizocarpa (B). (TIFF) [file pone.0017863.s001.tiff]

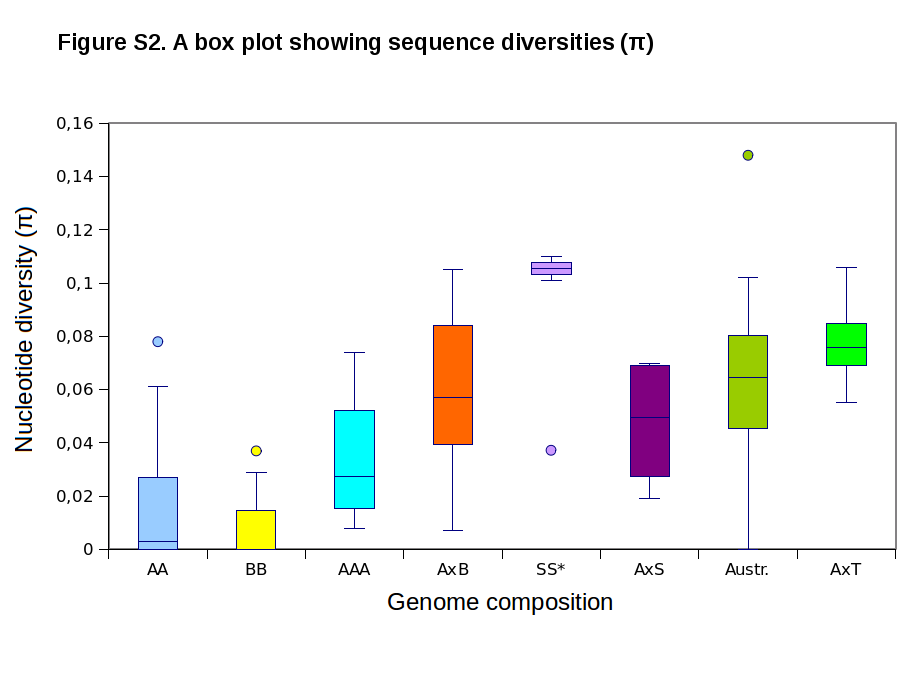

Supplement: Figure S2 — A box plot showing sequence diversities (π) within Musa representatives with different genome constitution and hybrid clones. The inter-subspecific diploids were not included in the analysis. Three diploid species which gave unexpected clustering in the phylogenetic tree (see Results and Discussion) were included in the data set according the results of NJ and BI analysis. * Only three representatives of S genome were included in our study. Two of them contained 3 ITS types including putative pseudogenic sequences and show the highest nucleotide difference among the analyzed species. (TIFF) [file pone.0017863.s002.tiff]

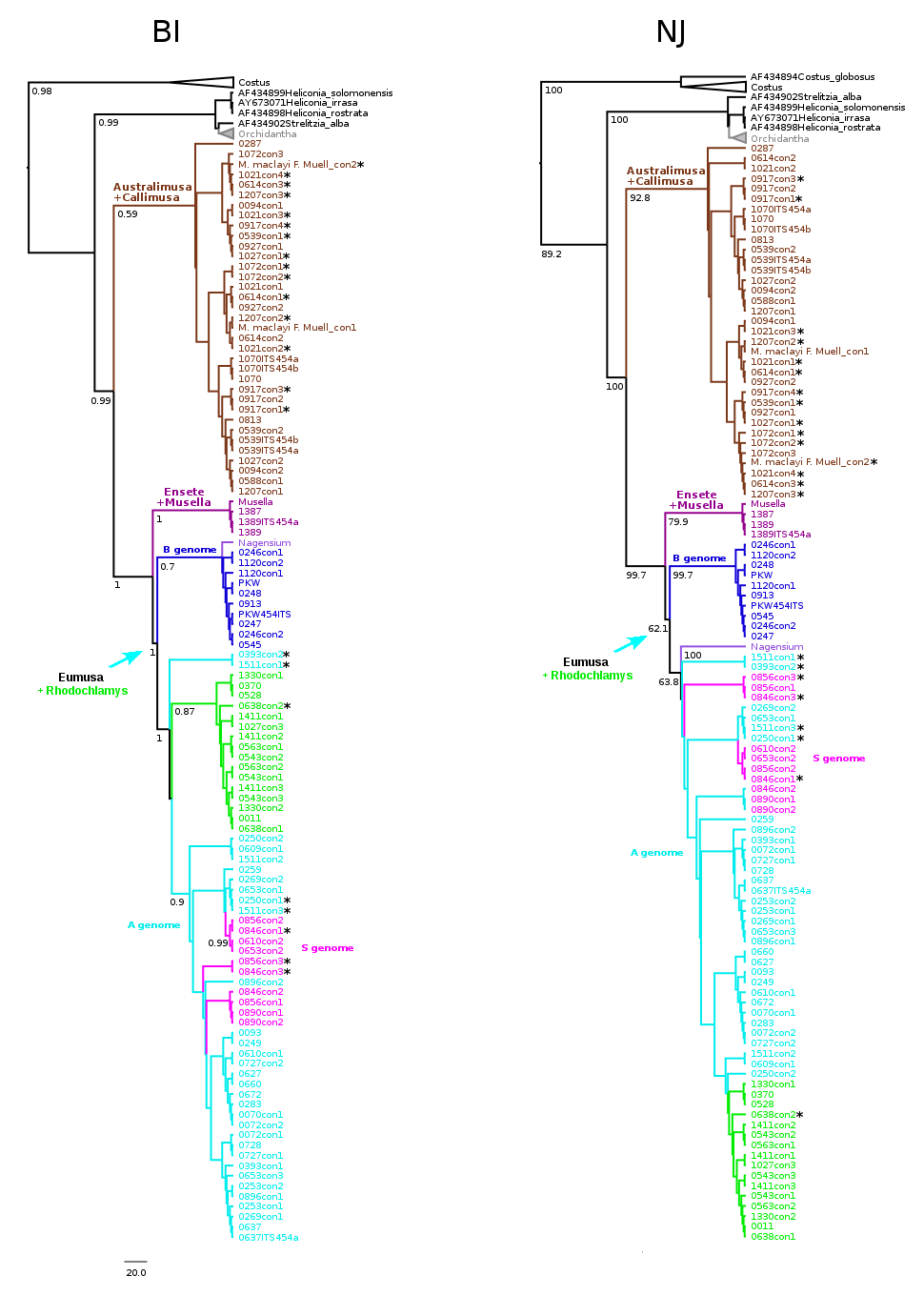

Supplement: Figure S3 — A BI and NJ phylogenetic trees of all ITS types including putative pseudogenes, obtained in diploid Musaceae accessions and selected species of closely related families Strelitziaceae, Lowiaceae, Heliconiaceae and Costaceae based on the ITS1-ITS2 sequence region. NJ tree was constructed from a Jukes-Cantor distance matrix and BI analysis of the same data set was performed in BEAST v1.5.3 using GTR+I+G model of nucleotide substitution. Values below the branches indicate the bootstrap support of NJ analysis and posterior probability of BI, respectively. Main clades and subclades are labeled by different colors as used in Figure 3 and putative pseudogenic types of ITS sequences are marked by asterisks. (TIFF) [file pone.0017863.s003.tiff]

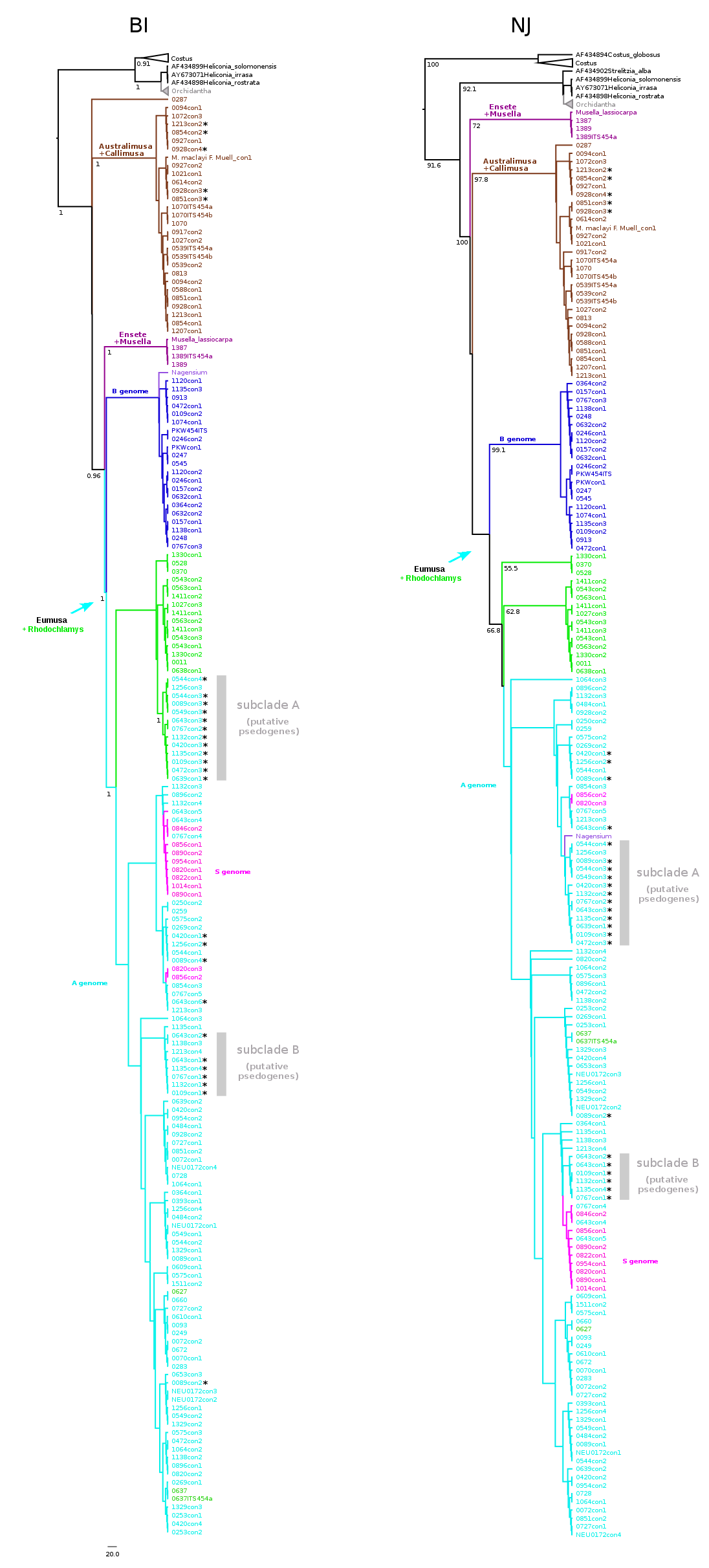

Supplement: Figure S4 — A BI and NJ phylogenetic analysis trees of all evaluated Musaceae accessions including banana hybrids and selected species of the closely related families Strelitziaceae, Lowiaceae, Heliconiaceae and Costaceae based on the ITS1-ITS2 sequence region. NJ tree was constructed from a Jukes-Cantor distance matrix and BI analysis of the same data set was performed in BEAST v1.5.3 using GTR+I+G model of nucleotide substitution. Values below the branches indicate the bootstrap support of NJ analysis and posterior probability of BI, respectively. Main clades and subclades are labeled by different colors as used in Figure 3 and putative pseudogenic types of ITS sequences of hybrids are marked by asterisks. (TIFF) [file pone.0017863.s004.tiff]
