# Supplementary material for: The ITS1-5.8S-ITS2 Sequence Region in the Musaceae: Structure, Diversity and Use in Molecular Phylogeny
Source: PLoS One. 2011 Mar 22;6(3):e17863. doi: 10.1371/journal.pone.0017863 (PMC3062550; doi:10.1371/journal.pone.0017863)
Supplement: Table S1 — Diploid representatives of the family Musaceae and Musa hybrid clones used in the study. Diploid representatives that are probably inter-subspecific hybrids [83] are marked by asterisk. (DOC) [file pone.0017863.s005.doc]

**Table S1: Diploid representatives of the family Musaceae**

| **Genus** |  | **Section** |  | **Sp./Group** |  | **Ssp./Subgroup** |  | **Accession name** |  | **ITC code** |  | **Number of sequences analyzed** |  | **Note **** |
| --- | --- | --- | --- | --- | --- | --- | --- | --- | --- | --- | --- | --- | --- | --- |
| ***Musa*** |  |  |  |  |  |  |  |  |  |  |  |  |  |  |
|  |  | Eumusa |  |  |  |  |  |  |  |  |  |  |  |  |
|  |  |  |  | acuminata |  | banksii |  | M. acuminata ssp. banksii |  | 0896 |  | 34 |  |  |
|  |  |  |  |  |  | burmannica |  | Tavoy |  | 0072 |  | 36 |  |  |
|  |  |  |  |  |  | burmannica |  | Long Tavoy |  | 0093 |  | Direct |  |  |
|  |  |  |  |  |  | burmannica |  | Long Tavoy |  | 0283 |  | Direct |  |  |
|  |  |  |  |  |  | burmannicoides |  | Calcutta4 |  | 0249 |  | Direct |  | **454** |
|  |  |  |  |  |  | malaccensis |  | Malaccensis |  | 0250 |  | 29 |  |  |
|  |  |  |  |  |  | malaccensis |  | Pahang IRFA |  | 0070 |  | 18 |  |  |
|  |  |  |  |  |  | malaccensis |  | Pahang |  | 0609 |  | 15 |  |  |
|  |  |  |  |  |  | malaccensis |  | Pahang |  | 0727 |  | 29 |  |  |
|  |  |  |  |  |  | malaccensis |  | DH Pahang |  | 1511 |  | 40 |  |  |
|  |  |  |  |  |  | microcarpa |  | Borneo |  | 0253 |  | 16 |  |  |
|  |  |  |  |  |  | siamea |  | Khae (Phrae) |  | 0660 |  | Direct |  |  |
|  |  |  |  |  |  | siamea |  | Pa (Rayong) |  | 0672 |  | Direct |  |  |
|  |  |  |  |  |  | truncata |  | Truncata |  | 0393 |  | 18 |  |  |
|  |  |  |  |  |  | zebrina |  | Maia Oa |  | 0728 |  | Direct |  |  |
|  |  |  |  |  |  | sucrier |  | Pisang Mas * |  | 0653 |  | 38 |  |  |
|  |  |  |  |  |  | --- |  | Galeo * |  | 0259 |  | Direct |  |  |
|  |  |  |  |  |  | --- |  | Niyarma yik * |  | 0269 |  | 40 |  |  |
|  |  |  |  |  |  | --- |  | Tuu Gia * |  | 0610 |  | 15 |  |  |
|  |  |  |  |  |  |  |  |  |  |  |  |  |  |  |
|  |  |  |  | schizocarpa |  |  |  | M. schizocarpa |  | 0846 |  | 59 |  |  |
|  |  |  |  |  |  |  |  | M. schizocarpa |  | 0856 |  | 42 |  |  |
|  |  |  |  |  |  |  |  | M. schizocarpa |  | 0890 |  | 15 |  |  |
|  |  |  |  |  |  |  |  |  |  |  |  |  |  |  |
|  |  |  |  | balbisiana |  |  |  | M. balbisiana (10852) |  | 0094 |  | 37 |  |  |
|  |  |  |  |  |  |  |  | Cameroun |  | 0246 |  | 15 |  |  |
|  |  |  |  |  |  |  |  | Honduras |  | 0247 |  | Direct |  |  |
|  |  |  |  |  |  |  |  | Singapuri |  | 0248 |  | Direct |  |  |
|  |  |  |  |  |  |  |  | M. balbisiana |  | 0545 |  | Direct |  |  |
|  |  |  |  |  |  |  |  | Tani |  | 1120 |  | 23 |  |  |
|  |  |  |  |  |  |  |  | Pisang Klutug Wulung |  | --- |  | Direct |  | **454** |
|  |  |  |  |  |  |  |  |  |  |  |  |  |  |  |
|  |  |  |  | nagensium |  |  |  | M. nagensium |  | --- |  | 15 |  |  |
|  |  |  |  |  |  |  |  |  |  |  |  |  |  |  |
|  |  | Rhodochlamys |  | laterita |  |  |  | M. laterita |  | 0627 |  | Direct |  |  |
|  |  |  |  |  |  |  |  |  |  |  |  |  |  |  |
|  |  |  |  | ornata |  |  |  | M. ornata |  | 0370 |  | Direct |  |  |
|  |  |  |  |  |  |  |  | M. ornata |  | 0637 |  | Direct |  | **454** |
|  |  |  |  |  |  |  |  | Kluai Bou |  | 0528 |  | Direct |  |  |
|  |  |  |  |  |  |  |  | M. ornata Red fingers |  | 1330 |  | 35 |  |  |
|  |  |  |  |  |  |  |  |  |  |  |  |  |  |  |
|  |  |  |  | sanguinea |  |  |  | M. mannii H. Wendl |  | 0543 |  | 36 |  |  |
|  |  |  |  |  |  |  |  | M. mannii |  | 1411 |  | 39 |  |  |
|  |  |  |  |  |  |  |  |  |  |  |  |  |  |  |
|  |  |  |  | velutina |  |  |  | M. velutina |  | 0011 |  | Direct |  |  |
|  |  |  |  |  |  |  |  | M. velutina |  | 0638 |  | 38 |  |  |
|  |  |  |  |  |  |  |  |  |  |  |  |  |  |  |
|  |  | Callimusa |  | beccarii |  |  |  | M. beccarii |  | 1070 |  | Direct |  | **454** |
|  |  |  |  |  |  |  |  |  |  |  |  |  |  |  |
|  |  |  |  | coccinea |  |  |  | M. coccinea |  | 0287 |  | Direct |  |  |
|  |  |  |  |  |  |  |  |  |  |  |  |  |  |  |
|  |  | Australimusa |  | Fe´i |  |  |  | Wain |  | 0813 |  | Direct |  |  |
|  |  |  |  |  |  |  |  | Utafan |  | 0913 |  | Direct |  |  |
|  |  |  |  |  |  |  |  | Kawaputa |  | 0927 |  | 47 |  |  |
|  |  |  |  |  |  |  |  | Menei |  | 1021 |  | 30 |  |  |
|  |  |  |  |  |  |  |  | Asupina |  | 1027 |  | 52 |  |  |
|  |  |  |  |  |  |  |  |  |  |  |  |  |  |  |
|  |  |  |  | jackeyi |  |  |  | M. jackeyi |  | 0588 |  | 18 |  |  |
|  |  |  |  |  |  |  |  |  |  |  |  |  |  |  |
|  |  |  |  | maclayi |  |  |  | M. maclayi type Hung Si |  | 0614 |  | 29 |  |  |
|  |  |  |  |  |  |  |  | M. maclayi |  | 1207 |  | 23 |  |  |
|  |  |  |  |  |  |  |  | M. maclayi F.Muell |  | --- |  | 39 |  |  |
|  |  |  |  |  |  |  |  |  |  |  |  |  |  |  |
|  |  |  |  | peekelii |  |  |  | M. peekelii ssp. peekelii |  | 0917 |  | 25 |  |  |
|  |  |  |  |  |  |  |  |  |  |  |  |  |  |  |
|  |  |  |  | textilis |  |  |  | M. textilis Née |  | 0563 |  | 47 |  |  |
|  |  |  |  |  |  |  |  | M. textilis |  | 0539 |  | 15 |  | **454** |
|  |  |  |  |  |  |  |  | M. textilis |  | 1072 |  | 29 |  |  |
|  |  |  |  |  |  |  |  |  |  |  |  |  |  |  |
| ***Ensete*** |  |  |  |  |  |  |  |  |  |  |  |  |  |  |
|  |  |  |  | ventricosum |  |  |  | Ensete ventricosum |  | 1387 |  | Direct |  |  |
|  |  |  |  | gilletii |  |  |  | Ensete gilletii |  | 1389 |  | Direct |  | **454** |
|  |  |  |  |  |  |  |  |  |  |  |  |  |  |  |
| ***Musella*** |  |  |  |  |  |  |  |  |  |  |  |  |  |  |
|  |  |  |  |  |  |  |  | Musella lasiocarpa |  | --- |  | Direct |  |  |
|  |  |  |  |  |  |  |  |  |  |  |  |  |  |  |

* Diploid representatives that are probably inter-subspecific hybrids [83].

** Sequenced by 454.

**Table S1: *Musa* hybrid clones used in the study**

| **Genus** |  | **Genome composition** |  | **Sp./Group** |  | **Accession name** |  | **ITC code** |  | **Number of sequences analyzed** |
| --- | --- | --- | --- | --- | --- | --- | --- | --- | --- | --- |
| ***Musa*** |  |  |  |  |  |  |  |  |  |  |
|  |  | AAA |  |  |  |  |  |  |  |  |
|  |  |  |  | Ambon |  | Pisang Bakar |  | 1064 |  | 46 |
|  |  |  |  |  |  |  |  |  |  |  |
|  |  |  |  | Cavendish |  | Grande Naine |  | --- (NEU0172) |  | 51 |
|  |  |  |  |  |  |  |  |  |  |  |
|  |  |  |  | Gros Michel |  | Gros Michel |  | 0484 |  | 63 |
|  |  |  |  |  |  |  |  |  |  |  |
|  |  |  |  | Red/Green Red |  | Red Dacca |  | 0575 |  | 56 |
|  |  |  |  |  |  |  |  |  |  |  |
|  |  |  |  | Orotava |  | Pisang Kayu |  | 0420 |  | 47 |
|  |  |  |  |  |  |  |  |  |  |  |
|  |  |  |  | --- |  | Gran Enano |  | 1256 |  | 59 |
|  |  |  |  | --- |  | Hochuchu |  | 0549 |  | 58 |
|  |  |  |  | --- |  | Not named M. paradisiaca |  | 0089 |  | 61 |
|  |  |  |  | --- |  | Not named M. paradisiaca x |  | 0544 |  | 54 |
|  |  |  |  | --- |  | Novaria |  | 1329 |  | 51 |
|  |  |  |  |  |  |  |  |  |  |  |
|  |  | AAB |  |  |  |  |  |  |  |  |
|  |  |  |  | Iholena |  | Maritú |  | 0639 |  | 45 |
|  |  |  |  |  |  |  |  |  |  |  |
|  |  |  |  | Plantain |  | Obino l'Ewai |  | 0109 |  | 57 |
|  |  |  |  |  |  | 3 Hands Planty |  | 1132 |  | 66 |
|  |  |  |  |  |  |  |  |  |  |  |
|  |  |  |  | Popoulou/Maia Maoli |  | Popoulou (CMR) |  | 1135 |  | 68 |
|  |  |  |  |  |  |  |  |  |  |  |
|  |  | ABB |  |  |  |  |  |  |  |  |
|  |  |  |  | Bluggoe |  | Cachaco |  | 0643 |  | 87 |
|  |  |  |  |  |  | Cachaco Enano |  | 0632 |  | 41 |
|  |  |  |  |  |  | Dole |  | 0767 |  | 73 |
|  |  |  |  |  |  | Kivuvu |  | 0157 |  | 42 |
|  |  |  |  |  |  | Silver Bluggoe |  | 0364 |  | 30 |
|  |  |  |  |  |  |  |  |  |  |  |
|  |  |  |  | Saba |  | Saba |  | 1138 |  | 55 |
|  |  |  |  |  |  |  |  |  |  |  |
|  |  |  |  | --- |  | Pelipita |  | 0472 |  | 77 |
|  |  |  |  |  |  |  |  |  |  |  |
|  |  | AS |  |  |  |  |  |  |  |  |
|  |  |  |  | --- |  | Ato |  | 0820 |  | 49 |
|  |  |  |  | --- |  | Tonton Kepa |  | 0822 |  | 56 |
|  |  |  |  | --- |  | Ungota |  | 0954 |  | 46 |
|  |  |  |  |  |  |  |  |  |  |  |
|  |  | AxS |  | --- |  | M. acuminata ssp. x M. schizocarpa |  | 1014 |  | 22 |
|  |  |  |  |  |  |  |  |  |  |  |
|  |  | AxT |  |  |  |  |  |  |  |  |
|  |  |  |  | --- |  | Karoina |  | 0851 |  | 57 |
|  |  |  |  | --- |  | Kabulupusa |  | 0928 |  | 54 |
|  |  |  |  | --- |  | Sar |  | 1213 |  | 47 |
|  |  |  |  | --- |  | Umbubu |  | 0854 |  | 45 |
|  |  |  |  |  |  |  |  |  |  |  |
|  |  | BxT |  |  |  |  |  |  |  |  |
|  |  |  |  | --- |  | Butuhan |  | 1074 |  | 22 |
|  |  |  |  |  |  |  |  |  |  |  |
