# Supplementary material for: The ITS1-5.8S-ITS2 Sequence Region in the Musaceae: Structure, Diversity and Use in Molecular Phylogeny
Source: PLoS One. 2011 Mar 22;6(3):e17863. doi: 10.1371/journal.pone.0017863 (PMC3062550; doi:10.1371/journal.pone.0017863)
Supplement: Table S2 — Sequence characteristics of ITS1-5.8S-ITS2 regions in Musaceae. (DOC) [file pone.0017863.s006.doc]

***Table S2. Sequence characteristics of ITS1-5.8S-ITS2 region in*** Musaceae

| **Accession name** |  | **Name of ITS type▲** | | |  | **GC content [%]** | | | | | | | |  | **Position of nucleotide changes**  **(nt-) in conserved 5.8S motives** | | | | | | | | | |  | | **Secondary structure of ITS2** | |  | **Secondary structure of 5.8S** |  | **Note** | | |
| --- | --- | --- | --- | --- | --- | --- | --- | --- | --- | --- | --- | --- | --- | --- | --- | --- | --- | --- | --- | --- | --- | --- | --- | --- | --- | --- | --- | --- | --- | --- | --- | --- | --- | --- |
|  |  |  |  |  |  | **ITS1** | |  | | **5.8S** |  | | **ITS2** |  | **Motif M1** | |  | | **Motif M2** | |  | | **Motif M3** | |  | |  |  |  |  |  |  |  |  |
| M. acuminata ssp. banksii |  | 0896con1 | | |  | 62.91 | |  | | 57.42 |  | | 70.78 |  | conserved | |  | | conserved | |  | | conserved | |  | | four-helices | |  | conserved |  |  | | |
|  |  | 0896con2 | | |  | 62.50 | |  | | 56.77 |  | | 68.22 |  | conserved | |  | | conserved | |  | | conserved | |  | | four-helices | |  | conserved |  |  | | |
|  |  |  | | |  |  | |  | |  |  | |  |  |  | |  | |  | |  | |  | |  | |  | |  |  |  |  | | |
| Tavoy |  | 0072con1 | | |  | 62.04 | |  | | 57.42 |  | | 69.44 |  | conserved | |  | | conserved | |  | | conserved | |  | | four-helices | |  | conserved |  |  | | |
|  |  | 0072con2 | | |  | 62.04 | |  | | 57.42 |  | | 69.16 |  | conserved | |  | | conserved | |  | | conserved | |  | | four-helices | |  | conserved |  |  | | |
|  |  |  | | |  |  | |  | |  |  | |  |  |  | |  | |  | |  | |  | |  | |  | |  |  |  |  | | |
| Long Tavoy |  | 0093 | | |  | 62.04 | |  | | 57.42 |  | | 68.69 |  | conserved | |  | | conserved | |  | | conserved | |  | | four-helices | |  | conserved |  |  | | |
|  |  |  | | |  |  | |  | |  |  | |  |  |  | |  | |  | |  | |  | |  | |  | |  |  |  |  | | |
| Long Tavoy |  | 0283 | | |  | 62.04 | |  | | 57.42 |  | | 69.16 |  | conserved | |  | | conserved | |  | | conserved | |  | | four-helices | |  | conserved |  |  | | |
|  |  |  | | |  |  | |  | |  |  | |  |  |  | |  | |  | |  | |  | |  | |  | |  |  |  |  | | |
| Calcutta4 |  | 0249 | | |  | 61.86 | |  | | 57.42 |  | | 68.69 |  | conserved | |  | | conserved | |  | | conserved | |  | | four-helices | |  | conserved |  |  | | |
|  |  | 0249ITS454-type1 | | |  | 61.57 | |  | | 57.42 |  | | 69.16 |  | conserved | |  | | conserved | |  | | conserved | |  | | four-helices | |  | conserved |  |  | | |
|  |  |  | | |  |  | |  | |  |  | |  |  |  | |  | |  | |  | |  | |  | |  | |  |  |  |  | | |
| Malaccensis |  | **0250con1** | | |  | **60.65** | |  | | **53.55** |  | | **62.15** |  | **conserved** | |  | | **conserved** | |  | | **conserved** | |  | | **not formed** | |  | **conserved** |  | **pseudogene** | | |
|  |  | 0250con2 | | |  | 62.96 | |  | | 57.42 |  | | 69.16 |  | conserved | |  | | conserved | |  | | conserved | |  | | four-helices | |  | conserved |  |  | | |
|  |  |  | | |  |  | |  | |  |  | |  |  |  | |  | |  | |  | |  | |  | |  | |  |  |  |  | | |
| Pahang IRFA |  | 0070con1 | | |  | 62.04 | |  | | 57.42 |  | | 69.16 |  | conserved | |  | | conserved | |  | | conserved | |  | | four-helices | |  | conserved |  |  | | |
|  |  |  | | |  |  | |  | |  |  | |  |  |  | |  | |  | |  | |  | |  | |  | |  |  |  |  | | |
| Pahang |  | 0609con1 | | |  | 63.59 | |  | | 57.42 |  | | 68.37 |  | conserved | |  | | conserved | |  | | conserved | |  | | four-helices | |  | conserved |  |  | | |
|  |  |  | | |  |  | |  | |  |  | |  |  |  | |  | |  | |  | |  | |  | |  | |  |  |  |  | | |
| Pahang |  | 0727con1 | | |  | 62.04 | |  | | 57.42 |  | | 69.77 |  | conserved | |  | | conserved | |  | | conserved | |  | | four-helices | |  | conserved |  |  | | |
|  |  | 0727con2 | | |  | 62.04 | |  | | 57.42 |  | | 68.69 |  | conserved | |  | | conserved | |  | | conserved | |  | | four-helices | |  | conserved |  |  | | |
|  |  |  | | |  |  | |  | |  |  | |  |  |  | |  | |  | |  | |  | |  | |  | |  |  |  |  | | |
| DH Pahang |  | **1511con1** | | |  | **59.26** | |  | | **55.48** |  | | **61.79** |  | **conserved** | |  | | **nt-9 “T”** | |  | | **conserved** | |  | | **not formed** | |  | **conserved** |  | **pseudogene** | | |
|  |  | 1511con2 | | |  | 63.13 | |  | | 57.42 |  | | 68.37 |  | conserved | |  | | conserved | |  | | conserved | |  | | four-helices | |  | conserved |  |  | | |
|  |  | **1511con3** | | |  | **59.72** | |  | | **53.25** |  | | **61.68** |  | **conserved** | |  | | **conserved** | |  | | **conserved** | |  | | **not formed** | |  | **not formed** |  | **pseudogene** | | |
|  |  |  | | |  |  | |  | |  |  | |  |  |  | |  | |  | |  | |  | |  | |  | |  |  |  |  | | |
| Borneo |  | 0253con1 | | |  | 62.33 | |  | | 57.42 |  | | 69.77 |  | conserved | |  | | conserved | |  | | conserved | |  | | four-helices | |  | conserved |  |  | | |
|  |  | 0253con2 | | |  | 62.44 | |  | | 57.69 |  | | 70.23 |  | conserved | |  | | conserved | |  | | conserved | |  | | four-helices | |  | conserved |  |  | | |
|  |  |  | | |  |  | |  | |  |  | |  |  |  | |  | |  | |  | |  | |  | |  | |  |  |  |  | | |
| Khae (Phrae) |  | 0660 | | |  | 61.57 | |  | | 57.42 |  | | 69.16 |  | conserved | |  | | conserved | |  | | conserved | |  | | four-helices | |  | conserved |  |  | | |
|  |  |  | | |  |  | |  | |  |  | |  |  |  | |  | |  | |  | |  | |  | |  | |  |  |  |  | | |
| Pa (Rayong) |  | 0672 | | |  | 62.04 | |  | | 57.42 |  | | 69.16 |  | conserved | |  | | conserved | |  | | conserved | |  | | four-helices | |  | conserved |  |  | | |
|  |  |  | | |  |  | |  | |  |  | |  |  |  | |  | |  | |  | |  | |  | |  | |  |  |  |  | | |
| Truncata |  | 0393con1 | | |  | 62.96 | |  | | 58.06 |  | | 68.98 |  | conserved | |  | | conserved | |  | | conserved | |  | | four-helices | |  | conserved |  |  | | |
|  |  | **0393con2** | | |  | **57.41** | |  | | **52.90** |  | | **63.77** |  | **nt-12 “A”** | |  | | **nt-9 “T”**  **nt-14 “T”** | |  | | **conserved** | |  | | **not formed** | |  | **not formed** |  | **pseudogene** | | |
|  |  |  | | |  |  | |  | |  |  | |  |  |  | |  | |  | |  | |  | |  | |  | |  |  |  |  | | |
| Maia Oa |  | 0728 | | |  | 61.40 | |  | | 57.42 |  | | 69.77 |  | conserved | |  | | conserved | |  | | conserved | |  | | four-helices | |  | conserved |  |  | | |
|  |  |  | | |  |  | |  | |  |  | |  |  |  | |  | |  | |  | |  | |  | |  | |  |  |  |  | | |
| M. schizocarpa |  | **0846con1** | | |  | **55.35** | |  | | **52.26** |  | | **62.15** |  | **conserved** | |  | | **conserved** | |  | | **conserved** | |  | | **not formed** | |  | **conserved** |  | **pseudogene** | | |
|  |  | 0846con2 | | |  | 62.04 | |  | | 57.42 |  | | 69.16 |  | conserved | |  | | conserved | |  | | conserved | |  | | four-helices | |  | conserved |  |  | | |
|  |  | **0846con3** | | |  | **62.50** | |  | | **54.19** |  | | **63.08** |  | **nt-12 “A”** | |  | | **conserved** | |  | | **conserved** | |  | | **four-helices** | |  | **not formed** |  | **pseudogene** | | |
|  |  |  | | |  |  | |  | |  |  | |  |  |  | |  | |  | |  | |  | |  | |  | |  |  |  |  | | |
| M. schizocarpa |  | 0856con1 | | |  | 61.11 | |  | | 56.77 |  | | 67.29 |  | conserved | |  | | conserved | |  | | conserved | |  | | four-helices | |  | conserved |  |  | | |
|  |  | 0856con2 | | |  | 59.72 | |  | | 54.19 |  | | 65.89 |  | conserved | |  | | conserved | |  | | conserved | |  | | four-helices | |  | conserved |  |  | | |
|  |  | **0856con3** | | |  | **56.94** | |  | | **50.97** |  | | **61.68** |  | **nt-12 “A”** | |  | | **nt-9 “A”** | |  | | **nt-7 “T”** | |  | | **not formed** | |  | **not formed** |  | **pseudogene** | | |
|  |  |  | | |  |  | |  | |  |  | |  |  |  | |  | |  | |  | |  | |  | |  | |  |  |  |  | | |
| M. schizocarpa |  | 0890con1 | | |  | 64.06 | |  | | 57.42 |  | | 68.22 |  | conserved | |  | | conserved | |  | | conserved | |  | | four-helices | |  | conserved |  |  | | |
|  |  | 0890con2 | | |  | 62.96 | |  | | 57.42 |  | | 69.44 |  | conserved | |  | | conserved | |  | | conserved | |  | | four-helices | |  | conserved |  |  | | |
|  |  |  | | |  |  | |  | |  |  | |  |  |  | |  | |  | |  | |  | |  | |  | |  | conserved |  |  | | |
| M. balbisiana (10852) |  | 0094con1 | | |  | 63.68 | |  | | 57.42 |  | | 67.32 |  | conserved | |  | | conserved | |  | | conserved | |  | | four-helices | |  | conserved |  |  | | |
|  |  | 0094con2 | | |  | 66.82 | |  | | 58.06 |  | | 67.80 |  | conserved | |  | | conserved | |  | | conserved | |  | | four-helices | |  | conserved |  |  | | |
|  |  |  | | |  |  | |  | |  |  | |  |  |  | |  | |  | |  | |  | |  | |  | |  |  |  |  | | |
| Cameroun |  | 0246con1 | | |  | 60.47 | |  | | 57.42 |  | | 70.09 |  | conserved | |  | | conserved | |  | | conserved | |  | | four-helices | |  | conserved |  |  | | |
|  |  | 0246con2 | | |  | 62.33 | |  | | 57.42 |  | | 70.97 |  | conserved | |  | | conserved | |  | | conserved | |  | | four-helices | |  | conserved |  |  | | |
|  |  |  | | |  |  | |  | |  |  | |  |  |  | |  | |  | |  | |  | |  | |  | |  |  |  |  | | |
| Honduras |  | 0247 | | |  | 62.33 | |  | | 57.42 |  | | 70.97 |  | conserved | |  | | conserved | |  | | conserved | |  | | four-helices | |  | conserved |  |  | | |
|  |  |  | | |  |  | |  | |  |  | |  |  |  | |  | |  | |  | |  | |  | |  | |  |  |  |  | | |
| Singapuri |  | 0248 | | |  | 61.75 | |  | | 57.42 |  | | 70.37 |  | conserved | |  | | conserved | |  | | conserved | |  | | four-helices | |  | conserved |  |  | | |
|  |  |  | | |  |  | |  | |  |  | |  |  |  | |  | |  | |  | |  | |  | |  | |  |  |  |  | | |
| M. balbisiana |  | 0545 | | |  | 62.33 | |  | | 57.42 |  | | 70.97 |  | conserved | |  | | conserved | |  | | conserved | |  | | four-helices | |  | conserved |  |  | | |
|  |  |  | | |  |  | |  | |  |  | |  |  |  | |  | |  | |  | |  | |  | |  | |  |  |  |  | | |
| Tani |  | 1120con1 | | |  | 62.33 | |  | | 57.42 |  | | 70.70 |  | conserved | |  | | conserved | |  | | conserved | |  | | four-helices | |  | conserved |  |  | | |
|  |  | 1120con2 | | |  | 61.86 | |  | | 57.42 |  | | 70.56 |  | conserved | |  | | conserved | |  | | conserved | |  | | four-helices | |  | conserved |  |  | | |
|  |  |  | | |  |  | |  | |  |  | |  |  |  | |  | |  | |  | |  | |  | |  | |  |  |  |  | | |
| Pisang Klutug Wulung |  | PKW | | |  | 62.33 | |  | | 57.42 |  | | 70.83 |  | conserved | |  | | conserved | |  | | conserved | |  | | four-helices | |  | conserved |  |  | | |
|  |  | PKW454ITS | | |  | 62.33 | |  | | 57.42 |  | | 70.97 |  | conserved | |  | | conserved | |  | | conserved | |  | | four-helices | |  | conserved |  |  | | |
|  |  |  | | |  |  | |  | |  |  | |  |  |  | |  | |  | |  | |  | |  | |  | |  |  |  |  | | |
| M. nagensium |  | Nagensium-con1 | | |  | 57.41 | |  | | 52.26 |  | | 60.75 |  | conserved | |  | | conserved | |  | | nt-7 “T”  nt-4 “A” | |  | | four-helices | |  | conserved |  |  | | |
|  |  |  | | |  |  | |  | |  |  | |  |  |  | |  | |  | |  | |  | |  | |  | |  |  |  |  | | |
| M. laterita |  | 0627 | | |  | 61.57 | |  | | 57.42 |  | | 69.16 |  | conserved | |  | | conserved | |  | | conserved | |  | | four-helices | |  | conserved |  |  | | |
|  |  |  | | |  |  | |  | |  |  | |  |  |  | |  | |  | |  | |  | |  | |  | |  |  |  |  | | |
| M. ornata |  | 0370 | | |  | 61.57 | |  | | 58.06 |  | | 69.77 |  | conserved | |  | | conserved | |  | | conserved | |  | | four-helices | |  | conserved |  |  | | |
|  |  |  | | |  |  | |  | |  |  | |  |  |  | |  | |  | |  | |  | |  | |  | |  |  |  |  | | |
| M. ornata |  | 0637 | | |  | 62.50 | |  | | 57.42 |  | | 69.63 |  | conserved | |  | | conserved | |  | | conserved | |  | | four-helices | |  | conserved |  |  | | |
|  |  | 0637ITS454 | | |  | 62.50 | |  | | 57.42 |  | | 69.63 |  | conserved | |  | | conserved | |  | | conserved | |  | | four-helices | |  | conserved |  |  | | |
|  |  |  | | |  |  | |  | |  |  | |  |  |  | |  | |  | |  | |  | |  | |  | |  |  |  |  | | |
| Kluai Bou |  | 0528 | | |  | 61.57 | |  | | 58.06 |  | | 69.77 |  | conserved | |  | | conserved | |  | | conserved | |  | | four-helices | |  | conserved |  |  | | |
| M. ornata Red fingers |  | 1330con1 | | |  | 62.21 | |  | | 56.77 |  | | 70.09 |  | conserved | |  | | conserved | |  | | conserved | |  | | four-helices | |  | conserved |  |  | | |
|  |  | 1330con2 | | |  | 62.39 | |  | | 57.42 |  | | 69.48 |  | conserved | |  | | conserved | |  | | conserved | |  | | four-helices | |  | conserved |  |  | | |
|  |  |  | | |  |  | |  | |  |  | |  |  |  | |  | |  | |  | |  | |  | |  | |  |  |  |  | | |
| M. mannii H. Wendl |  | 0543con1 | | |  | 62.84 | |  | | 57.42 |  | | 68.69 |  | conserved | |  | | conserved | |  | | conserved | |  | | four-helices | |  | conserved |  |  | | |
|  |  | 0543con2 | | |  | 62.84 | |  | | 57.42 |  | | 70.09 |  | conserved | |  | | conserved | |  | | conserved | |  | | four-helices | |  | conserved |  |  | | |
|  |  | 0543con3 | | |  | 62.84 | |  | | 57.42 |  | | 68.28 |  | conserved | |  | | conserved | |  | | conserved | |  | | four-helices | |  | conserved |  |  | | |
|  |  | | |  |  | |  |  | |  |  | |  |  | |  |  | |  | |  | | |  |  | | |  |  |  |  | | |  |
|  |  |  | | |  |  | |  | |  |  | |  |  |  | |  | |  | |  | |  | |  |  | | |  |  |  |  | | |
| **Table S2. Continued** | | | | | | | | | | | | | | | | | | | | | | | | | | | | | | | | | | |
|  | | | | | | | | | | | | | | | | | | | | | | | | | | | | | | | | | | |
| **Accession name** |  | | **Name of ITS type▲** | |  | **GC content** | | | | | | | |  | **Position of nucleotide changes**  **(nt-) in conserved 5.8S motives** | | | | | | | | | |  | | **Secondary structure of ITS2** | |  | **Secondary structure of 5.8S** |  | | **Note** | |
|  |  | |  |  |  | **ITS1** | | |  | **5.8S** |  | **ITS2** | |  | **Motif M1** | | |  | | **Motif M2** |  | **Motif M3** | | |  | |  |  |  |  |  |  |  |  |
| M. mannii |  | | 1411con1 | |  | 63.30 | | |  | 57.42 |  | 69.63 | |  | conserved | | |  | | conserved |  | conserved | | |  | | four-helices | |  | conserved |  | |  | |
|  |  | | 1411con2 | |  | 62.39 | | |  | 56.77 |  | 69.63 | |  | conserved | | |  | | conserved |  | conserved | | |  | | four-helices | |  | conserved |  | |  | |
|  |  | | 1411con3 | |  | 63.01 | | |  | 57.42 |  | 68.28 | |  | conserved | | |  | | conserved |  | conserved | | |  | | four-helices | |  | conserved |  | |  | |
|  |  | |  | |  |  | | |  |  |  |  | |  |  | | |  | |  |  |  | | |  | |  | |  |  |  | |  | |
| M. velutina |  | | 0011 | |  | 62.39 | | |  | 57.42 |  | 69.48 | |  | conserved | | |  | | conserved |  | conserved | | |  | | four-helices | |  | conserved |  | |  | |
|  |  | |  | |  |  | | |  |  |  |  | |  |  | | |  | |  |  |  | | |  | |  | |  |  |  | |  | |
| M. velutina |  | | 0638con1 | |  | 62.39 | | |  | 57.42 |  | 69.48 | |  | conserved | | |  | | conserved |  | conserved | | |  | | four-helices | |  | conserved |  | |  | |
|  |  | | **0638con2** | |  | **61.93** | | |  | **54.19** |  | **66.82** | |  | **conserved** | | |  | | **conserved** |  | **nt-8 “A”** | | |  | | **not formed** | |  | **conserved** |  | | **pseudogene** | |
|  |  | |  | |  |  | | |  |  |  |  | |  |  | | |  | |  |  |  | | |  | |  | |  |  |  | |  | |
| M. beccarii |  | | 1070 | |  | 65.74 | | |  | 58.06 |  | 65.37 | |  | conserved | | |  | | conserved |  | conserved | | |  | | four-helices | |  | conserved |  | |  | |
|  |  | | 1070ITS454a | |  | 65.32 | | |  | 58.06 |  | 65.37 | |  | conserved | | |  | | conserved |  | conserved | | |  | | four-helices | |  | conserved |  | |  | |
|  |  | | 1070ITS454b | |  | 65.77 | | |  | 58.06 |  | 65.85 | |  | conserved | | |  | | conserved |  | conserved | | |  | | four-helices | |  | conserved |  | |  | |
|  |  | |  | |  |  | | |  |  |  |  | |  |  | | |  | |  |  |  | | |  | |  | |  |  |  | |  | |
| M. coccinea |  | | 0287 | |  | 66.52 | | |  | 58.06 |  | 69.59 | |  | conserved | | |  | | conserved |  | conserved | | |  | | four-helices | |  | conserved |  | |  | |
|  |  | |  | |  |  | | |  |  |  |  | |  |  | | |  | |  |  |  | | |  | |  | |  |  |  | |  | |
| Wain |  | | 0813 | |  | 65.47 | | |  | 58.06 |  | 67.65 | |  | conserved | | |  | | conserved |  | conserved | | |  | | four-helices | |  | conserved |  | |  | |
|  |  | |  | |  |  | | |  |  |  |  | |  |  | | |  | |  |  |  | | |  | |  | |  |  |  | |  | |
| Utafan |  | | 0913 | |  | 62.33 | | |  | 57.42 |  | 70.97 | |  | conserved | | |  | | conserved |  | conserved | | |  | | four-helices | |  | conserved |  | |  | |
|  |  | |  | |  |  | | |  |  |  |  | |  |  | | |  | |  |  |  | | |  | |  | |  |  |  | |  | |
| Kawaputa |  | | 0927con1 | |  | 58.30 | | |  | 51.61 |  | 59.02 | |  | conserved | | |  | | conserved |  | nt-7 “T” | | |  | | four-helices* | |  | conserved |  | |  | |
|  |  | | 0927con2 | |  | 60.81 | | |  | 53.55 |  | 64.39 | |  | conserved | | |  | | conserved |  | nt-7 “T” | | |  | | four-helices* | |  | conserved |  | |  | |
|  |  | |  | |  |  | | |  |  |  |  | |  |  | | |  | |  |  |  | | |  | |  | |  |  |  | |  | |
| Menei |  | | 1021con1 | |  | 65.92 | | |  | 56.77 |  | 67.32 | |  | nt-11 “T” | | |  | | conserved |  | conserved | | |  | | four-helices | |  | conserved |  | |  | |
|  |  | | **1021con2** | |  | **60.99** | | |  | **54.84** |  | **61.95** | |  | **conserved** | | |  | | **conserved** |  | **conserved** | | |  | | **not formed** | |  | **not formed** |  | | **pseudogene** | |
|  |  | | **1021con3** | |  | **64.13** | | |  | **51.75** |  | **63.90** | |  | **conserved** | | |  | | **nt-6 “A”** |  | **conserved** | | |  | | **not formed** | |  | **not formed** |  | | **pseudogene** | |
|  |  | | **1021con4** | |  | **59.34** | | |  | **52.26** |  | **61.84** | |  | **conserved** | | |  | | **conserved** |  | **conserved** | | |  | | **not formed** | |  | **not formed** |  | | **pseudogene** | |
|  |  | |  | |  |  | | |  |  |  |  | |  |  | | |  | |  |  |  | | |  | |  | |  |  |  | |  | |
| Asupina |  | | **1027con1** | |  | **58.74** | | |  | **51.61** |  | **58.54** | |  | **conserved** | | |  | | **conserved** |  | **nt-7 “T”** | | |  | | **four-helices** | |  | **not formed** |  | | **pseudogene** | |
|  |  | | 1027con2 | |  | 65.92 | | |  | 58.06 |  | 68.63 | |  | conserved | | |  | | conserved |  | conserved | | |  | | four-helices | |  | conserved |  | |  | |
|  |  | | 1027con3 | |  | 63.30 | | |  | 57.42 |  | 69.63 | |  | conserved | | |  | | conserved |  | conserved | | |  | | four-helices | |  | conserved |  | |  | |
|  |  | |  | |  |  | | |  |  |  |  | |  |  | | |  | |  |  |  | | |  | |  | |  |  |  | |  | |
| M. jackeyi |  | | 0588con1 | |  | 66.82 | | |  | 58.06 |  | 67.80 | |  | conserved | | |  | | conserved |  | conserved | | |  | | four-helices | |  | conserved |  | |  | |
|  |  | |  | |  |  | | |  |  |  |  | |  |  | | |  | |  |  |  | | |  | |  | |  |  |  | |  | |
| M. maclayi type Hung Si |  | | **0614con1** | |  | **61.43** | | |  | **52.90** |  | **63.41** | |  | **nt-11 “T”**  **nt-16 “T”** | | |  | | **conserved** |  | **conserved** | | |  | | **not formed** | |  | **not formed** |  | | **pseudogene** | |
|  |  | | 0614con2 | |  | 64.13 | | |  | 57.42 |  | 66.34 | |  | conserved | | |  | | conserved |  | conserved | | |  | | four-helices | |  | conserved |  | |  | |
|  |  | | **0614con3** | |  | **59.34** | | |  | **53.55** |  | **61.35** | |  | **conserved** | | |  | | **nt-6 “A”** |  | **conserved** | | |  | | **not formed** | |  | **not formed** |  | | **pseudogene** | |
|  |  | |  | |  |  | | |  |  |  |  | |  |  | | |  | |  |  |  | | |  | |  | |  |  |  | |  | |
| M. maclayi |  | | 1207con1 | |  | 67.17 | | |  | 58.06 |  | 67.32 | |  | conserved | | |  | | conserved |  | conserved | | |  | | four-helices | |  | conserved |  | |  | |
|  |  | | **1207con2** | |  | **65.02** | | |  | **56.77** |  | **66.34** | |  | **nt-11 “T”** | | |  | | **conserved** |  | **conserved** | | |  | | **four-helices** | |  | **not formed** |  | | **pseudogene** | |
|  |  | | **1207con3** | |  | **59.34** | | |  | **53.55** |  | **61.35** | |  | **conserved** | | |  | | **nt-6 “A”** |  | **conserved** | | |  | | **not formed** | |  | **not formed** |  | | **pseudogene** | |
|  |  | |  | |  |  | | |  |  |  |  | |  |  | | |  | |  |  |  | | |  | |  | |  |  |  | |  | |
| M. peekelii ssp. peekelii |  | | **0917con1** | |  | **64.13** | | |  | **52.90** |  | **64.88** | |  | **nt-12 “A”** | | |  | | **conserved** |  | **nt-4 “A”** | | |  | | **four-helices** | |  | **not formed** |  | | **pseudogene** | |
|  |  | | 0917con2 | |  | 62.78 | | |  | 52.26 |  | 64.88 | |  | nt-1 “T”  nt-16 “T” | | |  | | conserved |  | nt-4 “A” | | |  | | four-helices | |  | conserved |  | |  | |
|  |  | | **0917con3** | |  | **65.02** | | |  | **54.19** |  | **63.41** | |  | **nt-11 “T”** | | |  | | **nt-7 “T”** |  | **nt-4 “C”** | | |  | | **not formed** | |  | **not formed** |  | | **pseudogene** | |
|  |  | | **0917con4** | |  | **57.85** | | |  | **51.61** |  | **63.41** | |  | **conserved** | | |  | | **conserved** |  | **conserved** | | |  | | **four-helices** | |  | **not formed** |  | | **pseudogene** | |
|  |  | |  | |  |  | | |  |  |  |  | |  |  | | |  | |  |  |  | | |  | |  | |  |  |  | |  | |
| M. textilis Née |  | | 0563con1 | |  | 62.39 | | |  | 57.42 |  | 70.09 | |  | conserved | | |  | | conserved |  | conserved | | |  | | four-helices | |  | conserved |  | |  | |
|  |  | | 0563con2 | |  | 62.84 | | |  | 57.42 |  | 69.63 | |  | conserved | | |  | | conserved |  | conserved | | |  | | four-helices | |  | conserved |  | |  | |
|  |  | |  | |  |  | | |  |  |  |  | |  |  | | |  | |  |  |  | | |  | |  | |  |  |  | |  | |
| M. textilis |  | | **0539con1** | |  | **56.50** | | |  | **50.97** |  | **59.02** | |  | **conserved** | | |  | | **conserved** |  | **conserved** | | |  | | **not formed** | |  | **conserved** |  | | **pseudogene** | |
|  |  | | 0539con2 | |  | 66.82 | | |  | 58.06 |  | 68.78 | |  | conserved | | |  | | conserved |  | conserved | | |  | | four-helices | |  | conserved |  | |  | |
|  |  | | 0539ITS454a | |  | 66.82 | | |  | 58.06 |  | 67.96 | |  | conserved | | |  | | conserved |  | conserved | | |  | | four-helices | |  | conserved |  | |  | |
|  |  | | 0539ITS454b | |  | 66.37 | | |  | 58.06 |  | 68.45 | |  | conserved | | |  | | conserved |  | conserved | | |  | | four-helices | |  | conserved |  | |  | |
|  |  | |  | |  |  | | |  |  |  |  | |  |  | | |  | |  |  |  | | |  | |  | |  |  |  | |  | |
| M. textilis |  | | **1072con1** | |  | **63.68** | | |  | **50.97** |  | **61.39** | |  | **nt-2 “A”**  **nt-11 “T”**  **nt-14 “T”** | | |  | | **conserved** |  | **conserved** | | |  | | **not formed** | |  | **not formed** |  | | **pseudogene** | |
|  |  | | **1072con2** | |  | **62.16** | | |  | **53.25** |  | **61.35** | |  | **conserved** | | |  | | **nt-6 “T”**  **nt-9 “A”** |  | **conserved** | | |  | | **four-helices** | |  | **not formed** |  | | **pseudogene** | |
|  |  | | 1072con3 | |  | 58.79 | | |  | 54.19 |  | 62.32 | |  | nt-11 “T” | | |  | | nt-6 “A” |  | conserved | | |  | | four-helices | |  | conserved |  | |  | |
|  |  | |  | |  |  | | |  |  |  |  | |  |  | | |  | |  |  |  | | |  | |  | |  |  |  | |  | |
| M. maclayi F. Muell |  | | M. maclayi F. Muell_con1 | |  | 65.02 | | |  | 57.42 |  | 67.32 | |  | conserved | | |  | | conserved |  | conserved | | |  | | four-helices | |  | conserved |  | |  | |
|  |  | | **M. maclayi F. Muell_con2** | |  | **58.33** | | |  | **51.61** |  | **59.90** | |  | **conserved** | | |  | | **conserved** |  | **conserved** | | |  | | **not formed** | |  | **not formed** |  | | **pseudogene** | |
|  |  | |  | |  |  | | |  |  |  |  | |  |  | | |  | |  |  |  | | |  | |  | |  |  |  | |  | |
| Ensete ventricosum |  | | 1387 | |  | 61.11 | | |  | 57.42 |  | 66.98 | |  | conserved | | |  | | conserved |  | conserved | | |  | | four-helices | |  | conserved |  | |  | |
|  |  | |  | |  |  | | |  |  |  |  | |  |  | | |  | |  |  |  | | |  | |  | |  |  |  | |  | |
| Ensete gilletii |  | | 1389 | |  | 60.19 | | |  | 57.42 |  | 65.77 | |  | conserved | | |  | | conserved |  | conserved | | |  | | four-helices | |  | conserved |  | |  | |
|  |  | | 1389ITS454 | |  | 60.19 | | |  | 57.42 |  | 65.77 | |  | conserved | | |  | | conserved |  | conserved | | |  | | four-helices | |  | conserved |  | |  | |
|  |  | |  | |  |  | | |  |  |  |  | |  |  | | |  | |  |  |  | | |  | |  | |  |  |  | |  | |
| Musella lasiocarpa |  | | Musella | |  | 61.75 | | |  | 57.42 |  | 63.21 | |  | conserved | | |  | | conserved |  | conserved | | |  | | four-helices | |  | conserved |  | |  | |
|  |  | |  | |  |  | | |  |  |  |  | |  |  | | |  | |  |  |  | | |  | |  | |  |  |  | |  | |

| **Table S2. Continued** | | | | | | | | | | | | | | | | | | | | |
| --- | --- | --- | --- | --- | --- | --- | --- | --- | --- | --- | --- | --- | --- | --- | --- | --- | --- | --- | --- | --- |
|  | | | | | | | | | | | | | | | | | | | | |
| **Accession name** |  | **Name of ITS type▲** |  | **GC content** | | | | |  | **Position of nucleotide changes**  **(nt-) in conserved 5.8S motives** | | | | |  | **Secondary structure of ITS2** |  | **Secondary structure of 5.8S** |  | **Note** |
|  |  |  |  | **ITS1** |  | **5.8S** |  | **ITS2** |  | **Motif M1** |  | **Motif M2** |  | **Motif M3** |  |  |  |  |  |  |
| Pisang Mas |  | **0653con1** |  | **61.11** |  | **52.60** |  | **61.21** |  | **conserved** |  | **conserved** |  | **conserved** |  | **not formed** |  | **not formed** |  | **pseudogene** |
|  |  | **0653con2** |  | **57.87** |  | **54.19** |  | **63.55** |  | **nt-11 “T”**  **nt-16 “T”** |  | **nt-14 “T”** |  | **conserved** |  | **not formed** |  | **conserved** |  | **pseudogene** |
|  |  | 0653con3 |  | 62.50 |  | 57.42 |  | 69.91 |  | conserved |  | conserved |  | conserved |  | four-helices |  | conserved |  |  |
|  |  |  |  |  |  |  |  |  |  |  |  |  |  |  |  |  |  |  |  |  |
| Galeo |  | 0259 |  | 61.64 |  | 55.84 |  | 69.30 |  | conserved |  | conserved |  | conserved |  | four-helices |  | conserved |  |  |
|  |  |  |  |  |  |  |  |  |  |  |  |  |  |  |  |  |  |  |  |  |
| Niyarma yik |  | 0269con1 |  | 62.44 |  | 57.42 |  | 70.23 |  | conserved |  | conserved |  | conserved |  | four-helices |  | conserved |  |  |
|  |  | 0269con2 |  | 61.57 |  | 54.84 |  | 68.69 |  | conserved |  | conserved |  | conserved |  | four-helices |  | conserved |  |  |
|  |  |  |  |  |  |  |  |  |  |  |  |  |  |  |  |  |  |  |  |  |
| Tuu Gia |  | 0610con1 |  | 62.04 |  | 57.42 |  | 69.16 |  | conserved |  | conserved |  | conserved |  | four-helices |  | conserved |  |  |
|  |  | **0610con2** |  | **57.87** |  | **54.19** |  | **63.55** |  | **nt-11 “T”**  **nt-16 “T”** |  | **nt-14 “T”** |  | **conserved** |  | **not formed** |  | **not formed** |  | **pseudogene** |
|  |  |  |  |  |  |  |  |  |  |  |  |  |  |  |  |  |  |  |  |  |
| Pisang Bakar |  | 1064con1 |  | 61.57 |  | 57.42 |  | 69.77 |  | conserved |  | conserved |  | conserved |  | four-helices |  | conserved |  |  |
|  |  | 1064con2 |  | 62.91 |  | 57.42 |  | 70.78 |  | conserved |  | conserved |  | conserved |  | four-helices |  | conserved |  |  |
|  |  | 1064con3 |  | 63.59 |  | 57.42 |  | 69.77 |  | conserved |  | conserved |  | conserved |  | four-helices |  | conserved |  |  |
|  |  |  |  |  |  |  |  |  |  |  |  |  |  |  |  |  |  |  |  |  |
| Grande Naine |  | NEU0172con1 |  | 63.59 |  | 57.42 |  | 68.84 |  | conserved |  | conserved |  | conserved |  | four-helices |  | conserved |  |  |
|  |  | NEU0172con2 |  | 62.50 |  | 57.42 |  | 69.44 |  | conserved |  | conserved |  | conserved |  | four-helices* |  | conserved |  |  |
|  |  | NEU0172con3 |  | 62.50 |  | 57.42 |  | 69.72 |  | conserved |  | conserved |  | conserved |  | four-helices* |  | conserved |  |  |
|  |  | NEU0172con4 |  | 62.04 |  | 57.42 |  | 70.05 |  | conserved |  | conserved |  | conserved |  | four-helices |  | conserved |  |  |
|  |  |  |  |  |  |  |  |  |  |  |  |  |  |  |  |  |  |  |  |  |
| Gros Michel |  | 0484con1 |  | 62.04 |  | 57.42 |  | 68.84 |  | conserved |  | conserved |  | conserved |  | four-helices |  | conserved |  |  |
|  |  | 0484con2 |  | 63.59 |  | 57.42 |  | 68.84 |  | conserved |  | conserved |  | conserved |  | four-helices |  | conserved |  |  |
|  |  |  |  |  |  |  |  |  |  |  |  |  |  |  |  |  |  |  |  |  |
| Red Dacca |  | 0575con1 |  | 62.67 |  | 57.42 |  | 68.37 |  | conserved |  | conserved |  | conserved |  | four-helices |  | conserved |  |  |
|  |  | 0575con2 |  | 60.19 |  | 57.42 |  | 68.37 |  | conserved |  | conserved |  | conserved |  | four-helices |  | conserved |  |  |
|  |  | 0575con3 |  | 62.91 |  | 57.42 |  | 70.78 |  | conserved |  | conserved |  | conserved |  | four-helices |  | conserved |  |  |
|  |  |  |  |  |  |  |  |  |  |  |  |  |  |  |  |  |  |  |  |  |
| Pisang Kayu |  | **0420con1** |  | **58.33** |  | **52.60** |  | **64.49** |  | **nt-12 “T”** |  | **nt-6 “A”** |  | **conserved** |  | **not formed** |  | **not formed** |  | **pseudogene** |
|  |  | 0420con2 |  | 62.50 |  | 57.42 |  | 69.30 |  | conserved |  | conserved |  | conserved |  | four-helices |  | conserved |  |  |
|  |  | **0420con3** |  | **56.48** |  | **52.26** |  | **63.21** |  | **nt-16 “T”** |  | **nt-9 “T”** |  | **conserved** |  | **not formed** |  | **conserved** |  | **pseudogene** |
|  |  | 0420con4 |  | 62.50 |  | 57.42 |  | 70.37 |  | conserved |  | conserved |  | conserved |  | four-helices |  | conserved |  |  |
|  |  |  |  |  |  |  |  |  |  |  |  |  |  |  |  |  |  |  |  |  |
| Gran Enano |  | 1256con1 |  | 63.59 |  | 57.42 |  | 67.59 |  | conserved |  | conserved |  | conserved |  | four-helices |  | conserved |  |  |
|  |  | **1256con2** |  | **61.57** |  | **53.25** |  | **61.21** |  | **conserved** |  | **conserved** |  | **conserved** |  | **not formed** |  | **not formed** |  | **pseudogene** |
|  |  | 1256con3 |  | 59.72 |  | 55.49 |  | 62.56 |  | conserved |  | nt-9 “T” |  | conserved |  | four-helices |  | conserved |  |  |
|  |  | 1256con4 |  | 63.59 |  | 57.42 |  | 68.84 |  | conserved |  | conserved |  | conserved |  | four-helices |  | conserved |  |  |
|  |  |  |  |  |  |  |  |  |  |  |  |  |  |  |  |  |  |  |  |  |
| Hochuchu |  | 0549con1 |  | 63.59 |  | 57.42 |  | 68.84 |  | conserved |  | conserved |  | conserved |  | four-helices |  | conserved |  |  |
|  |  | 0549con2 |  | 62.67 |  | 58.06 |  | 69.44 |  | conserved |  | conserved |  | conserved |  | four-helices |  | conserved |  |  |
|  |  | **0549con3** |  | **57.60** |  | **55.48** |  | **61.79** |  | **conserved** |  | **nt-9 “T”** |  | **conserved** |  | **four-helices** |  | **conserved** |  | **pseudogene** |
|  |  |  |  |  |  |  |  |  |  |  |  |  |  |  |  |  |  |  |  |  |
| Not named  M. paradisiaca |  | 0089con1 |  | 63.59 |  | 57.42 |  | 68.84 |  | conserved |  | conserved |  | conserved |  | four-helices |  | conserved |  |  |
|  |  | **0089con2** |  | **62.50** |  | **57.69** |  | **69.44** |  | **conserved** |  | **conserved** |  | **conserved** |  | **four-helices** |  | **not formed** |  | **pseudogene** |
|  |  | **0089con3** |  | **57.41** |  | **54.84** |  | **61.79** |  | **conserved** |  | **nt-9 “T”**  **nt-14 “T”** |  | **conserved** |  | **not formed** |  | **not formed** |  | **pseudogene** |
|  |  | **0089con4** |  | **60.19** |  | **53.55** |  | **61.21** |  | **conserved** |  | **conserved** |  | **conserved** |  | **four-helices** |  | **not formed** |  | **pseudogene** |
|  |  |  |  |  |  |  |  |  |  |  |  |  |  |  |  |  |  |  |  |  |
| Not named  M. paradisiaca x |  | 0544con1 |  | 60.19 |  | 53.25 |  | 61.21 |  | conserved |  | conserved |  | conserved |  | four-helices |  | conserved |  |  |
|  |  | 0544con2 |  | 63.59 |  | 57.42 |  | 68.84 |  | conserved |  | conserved |  | conserved |  | four-helices |  | conserved |  |  |
|  |  | **0544con3** |  | **57.41** |  | **54.84** |  | **61.79** |  | **conserved** |  | **nt-9 “T”**  **nt-14 “T”** |  | **conserved** |  | **not formed** |  | **conserved** |  | **pseudogene** |
|  |  | **0544con4** |  | **58.33** |  | **54.19** |  | **62.26** |  | **conserved** |  | **nt-9 “T”** |  | **conserved** |  | **not formed** |  | **conserved** |  | **pseudogene** |
|  |  |  |  |  |  |  |  |  |  |  |  |  |  |  |  |  |  |  |  |  |
| Novaria |  | 1329con1 |  | 63.59 |  | 57.42 |  | 68.84 |  | conserved |  | conserved |  | conserved |  | four-helices |  | conserved |  |  |
|  |  | 1329con2 |  | 62.50 |  | 57.42 |  | 69.44 |  | conserved |  | conserved |  | conserved |  | four-helice* |  | conserved |  |  |
|  |  | 1329con3 |  | 62.50 |  | 57.42 |  | 70.09 |  | conserved |  | conserved |  | conserved |  | four-helices |  | conserved |  |  |
|  |  |  |  |  |  |  |  |  |  |  |  |  |  |  |  |  |  |  |  |  |
| Maritú |  | **0639con1** |  | **59.26** |  | **52.26** |  | **63.21** |  | **nt-16 “T”** |  | **nt-9 “T”** |  | **conserved** |  | **not formed** |  | **conserved** |  | **pseudogene** |
|  |  | 0639con2 |  | 62.50 |  | 57.42 |  | 70.05 |  | conserved |  | conserved |  | conserved |  | four-helices |  | conserved |  |  |
|  |  |  |  |  |  |  |  |  |  |  |  |  |  |  |  |  |  |  |  |  |
| Obino l'Ewai |  | **0109con1** |  | **56.94** |  | **52.26** |  | **64.02** |  | **nt-12 “A”** |  | **conserved** |  | **conserved** |  | **not formed** |  | **not formed** |  | **pseudogene** |
|  |  | 0109con2 |  | 62.33 |  | 57.42 |  | 70.97 |  | conserved |  | conserved |  | conserved |  | four-helices |  | conserved |  |  |
|  |  | **0109con3** |  | **59.26** |  | **52.26** |  | **63.21** |  | **nt-16 “T”** |  | **nt-9 “T”** |  | **conserved** |  | **not formed** |  | **conserved** |  | **pseudogene** |
|  |  |  |  |  |  |  |  |  |  |  |  |  |  |  |  |  |  |  |  |  |
| 3 Hands Planty |  | **1132con1** |  | **56.94** |  | **52.26** |  | **64.02** |  | **nt-12 “A”** |  | **conserved** |  | **conserved** |  | **not formed** |  | **not formed** |  | **pseudogene** |
|  |  | **1132con2** |  | **59.26** |  | **52.26** |  | **63.21** |  | **nt-16 “T”** |  | **nt-9 “T”** |  | **conserved** |  | **not formed** |  | **conserved** |  | **pseudogene** |
|  |  | 1132con3 |  | 61.11 |  | 52.90 |  | 66.36 |  | conserved |  | conserved |  | nt-6 “T” |  | four-helices |  | conserved |  |  |
|  |  | 1132con4 |  | 62.91 |  | 57.42 |  | 70.05 |  | conserved |  | conserved |  | conserved |  | four-helices |  | conserved |  |  |
|  |  |  |  |  |  |  |  |  |  |  |  |  |  |  |  |  |  |  |  |  |
| Popoulou (CMR) |  | 1135con1 |  | 62.50 |  | 57.42 |  | 70.23 |  | conserved |  | conserved |  | conserved |  | four-helices |  | conserved |  |  |
|  |  | **1135con2** |  | **59.26** |  | **52.26** |  | **63.21** |  | **nt-16 “T”** |  | **nt-9 “T”** |  | **conserved** |  | **not formed** |  | **conserved** |  | **pseudogene** |
|  |  | 1135con3 |  | 62.33 |  | 57.42 |  | 70.97 |  | conserved |  | conserved |  | conserved |  | four-helices |  | conserved |  |  |
|  |  | **1135con4** |  | **56.94** |  | **52.26** |  | **64.02** |  | **nt-12 “A”** |  | **conserved** |  | **conserved** |  | **not formed** |  | **not formed** |  | **pseudogene** |
|  |  |  |  |  |  |  |  |  |  |  |  |  |  |  |  |  |  |  |  |  |
| Cachaco Enano |  | 0632con1 |  | 62.33 |  | 57.42 |  | 70.70 |  | conserved |  | conserved |  | conserved |  | four-helices |  | conserved |  |  |
|  |  | 0632con2 |  | 62.33 |  | 57.42 |  | 70.78 |  | conserved |  | conserved |  | conserved |  | four-helices |  | conserved |  |  |
|  |  |  |  |  |  |  |  |  |  |  |  |  |  |  |  |  |  |  |  |  |

| **Table S2. Continued** | | | | | | | | | | | | | | | | | | | | |
| --- | --- | --- | --- | --- | --- | --- | --- | --- | --- | --- | --- | --- | --- | --- | --- | --- | --- | --- | --- | --- |
|  | | | | | | | | | | | | | | | | | | | | |
| **Accession name** |  | **Name of ITS type▲** |  | **GC content** | | | | |  | **Position of nucleotide changes**  **(nt-) in conserved 5.8S motives** | | | | |  | **Secondary structure of ITS2** |  | **Secondary structure of 5.8S** |  | **Note** |
|  |  |  |  | **ITS1** |  | **5.8S** |  | **ITS2** |  | **Motif M1** |  | **Motif M2** |  | **Motif M3** |  |  |  |  |  |  |
| Cachaco |  | **0643con1** |  | **56.94** |  | **52.90.** |  | **64.49** |  | **nt-12 “A”** |  | **conserved** |  | **conserved** |  | **not formed** |  | **not formed** |  | **pseudogene** |
|  |  | **0643con2** |  | **56.48** |  | **56.77** |  | **64.10** |  | **conserved** |  | **conserved** |  | **nt-9 “A”** |  | **not formed** |  | **conserved** |  | **pseudogene** |
|  |  | **0643con3** |  | **58.80** |  | **52.26** |  | **62.26** |  | **nt-16 “T”** |  | **nt-9 “T”** |  | **conserved** |  | **not formed** |  | **conserved** |  | **pseudogene** |
|  |  | 0643con4 |  | 62.04 |  | 57.42 |  | 69.77 |  | conserved |  | conserved |  | conserved |  | four-helices |  | conserved |  |  |
|  |  | 0643con5 |  | 63.89 |  | 56.77 |  | 68.98 |  | conserved |  | conserved |  | conserved |  | four-helices |  | conserved |  |  |
|  |  | **0643con6** |  | **57.87** |  | **54.19** |  | **64.09** |  | **nt-11 “T”**  **nt-16 “T”** |  | **nt-14 “T”** |  | **conserved** |  | **not formed** |  | **not formed** |  | **pseudogene** |
|  |  |  |  |  |  |  |  |  |  |  |  |  |  |  |  |  |  |  |  |  |
| Dole |  | **0767con1** |  | **56.94** |  | **52.26** |  | **64.02** |  | **nt-12 “A”** |  | **conserved** |  | **conserved** |  | **not formed** |  | **not formed** |  | **pseudogene** |
|  |  | **0767con2** |  | **58.80** |  | **52.26** |  | **62.26** |  | **conserved** |  | **nt-9 “T”** |  | **conserved** |  | **not formed** |  | **conserved** |  | **pseudogene** |
|  |  | 0767con3 |  | 62.33 |  | 57.42 |  | 70.70 |  | conserved |  | conserved |  | conserved |  | four-helices |  | conserved |  |  |
|  |  | 0767con4 |  | 62.96 |  | 57.42 |  | 69.77 |  | conserved |  | conserved |  | conserved |  | four-helices |  | conserved |  |  |
|  |  | 0767con5 |  | 60.19 |  | 56.77 |  | 67.43 |  | conserved |  | conserved |  | conserved |  | four-helices |  | conserved |  |  |
|  |  |  |  |  |  |  |  |  |  |  |  |  |  |  |  |  |  |  |  |  |
| Kivuvu |  | 0157con1 |  | 62.33 |  | 57.42 |  | 70.70 |  | conserved |  | conserved |  | conserved |  | four-helices |  | conserved |  |  |
|  |  | 0157con2 |  | 62.33 |  | 57.42 |  | 70.70 |  | conserved |  | conserved |  | conserved |  | four-helices |  | conserved |  |  |
|  |  |  |  |  |  |  |  |  |  |  |  |  |  |  |  |  |  |  |  |  |
| Silver Bluggoe |  | 0364con1 |  | 63.08 |  | 57.42 |  | 70.78 |  | conserved |  | conserved |  | conserved |  | four-helices |  | conserved |  |  |
|  |  | 0364con2 |  | 62.33 |  | 57.42 |  | 70.70 |  | conserved |  | conserved |  | conserved |  | four-helices |  | conserved |  |  |
|  |  |  |  |  |  |  |  |  |  |  |  |  |  |  |  |  |  |  |  |  |
| Saba |  | 1138con1 |  | 62.33 |  | 57.42 |  | 70.70 |  | conserved |  | conserved |  | conserved |  | four-helices |  | conserved |  |  |
|  |  | 1138con2 |  | 62.91 |  | 56.49 |  | 70.64 |  | conserved |  | conserved |  | conserved |  | four-helices |  | conserved |  |  |
|  |  | 1138con3 |  | 62.96 |  | 56.13 |  | 69.30 |  | conserved |  | nt-9 “T” |  | conserved |  | four-helices |  | conserved |  |  |
|  |  |  |  |  |  |  |  |  |  |  |  |  |  |  |  |  |  |  |  |  |
| Pelipita |  | 0472con1 |  | 62.33 |  | 57.42 |  | 71.30 |  | conserved |  | conserved |  | conserved |  | four-helices |  | conserved |  |  |
|  |  | 0472con2 |  | 62.91 |  | 57.42 |  | 70.64 |  | conserved |  | conserved |  | conserved |  | four-helices |  | conserved |  |  |
|  |  | **0472con3** |  | **59.26** |  | **52.26** |  | **63.21** |  | **nt-16 “T”** |  | **nt-9 “T”** |  | **conserved** |  | **not formed** |  | **conserved** |  | **pseudogene** |
|  |  |  |  |  |  |  |  |  |  |  |  |  |  |  |  |  |  |  |  |  |
| Ato |  | 0820con1 |  | 64.22 |  | 57.42 |  | 68.22 |  | conserved |  | conserved |  | conserved |  | four-helices |  | conserved |  |  |
|  |  | 0820con2 |  | 62.91 |  | 56.77 |  | 71.04 |  | conserved |  | conserved |  | conserved |  | four-helices |  | conserved |  |  |
|  |  | 0820con3 |  | 59.26 |  | 54.84 |  | 67.29 |  | conserved |  | conserved |  | conserved |  | four-helices |  | conserved |  |  |
|  |  |  |  |  |  |  |  |  |  |  |  |  |  |  |  |  |  |  |  |  |
| Tonton Kepa |  | 0822con1 |  | 63.59 |  | 57.42 |  | 68.81 |  | conserved |  | conserved |  | conserved |  | four-helices |  | conserved |  |  |
|  |  |  |  |  |  |  |  |  |  |  |  |  |  |  |  |  |  |  |  |  |
| Ungota |  | 0954con1 |  | 64.06 |  | 57.42 |  | 68.69 |  | conserved |  | conserved |  | conserved |  | four-helices |  | conserved |  |  |
|  |  | 0954con2 |  | 62.50 |  | 57.42 |  | 69.30 |  | conserved |  | conserved |  | conserved |  | four-helices |  | conserved |  |  |
|  |  |  |  |  |  |  |  |  |  |  |  |  |  |  |  |  |  |  |  |  |
| M. acuminata ssp. x M. schizocarpa |  | 1014con1 |  | 64.06 |  | 57.42 |  | 68.22 |  | conserved |  | conserved |  | conserved |  | four-helices |  | conserved |  |  |
|  |  |  |  |  |  |  |  |  |  |  |  |  |  |  |  |  |  |  |  |  |
|  |  |  |  |  |  |  |  |  |  |  |  |  |  |  |  |  |  |  |  |  |
| M. jackeyi |  | 0851con1 |  | 66.82 |  | 58.06 |  | 67.80 |  | conserved |  | conserved |  | conserved |  | four-helices |  | conserved |  |  |
|  |  | 0851con2 |  | 62.04 |  | 57.42 |  | 70.05 |  | conserved |  | conserved |  | conserved |  | four-helices |  | conserved |  |  |
|  |  | **0851con3** |  | **63.23** |  | **56.77** |  | **67.80** |  | **nt-11 “T”** |  | **conserved** |  | **conserved** |  | **four-helices** |  | **not formed** |  | **pseudogene** |
|  |  |  |  |  |  |  |  |  |  |  |  |  |  |  |  |  |  |  |  |  |
| Kabulupusa |  | 0928con1 |  | 66.82 |  | 58.06 |  | 67.80 |  | conserved |  | conserved |  | conserved |  | four-helices |  | conserved |  |  |
|  |  | 0928con2 |  | 62.04 |  | 57.42 |  | 70.32 |  | conserved |  | conserved |  | conserved |  | four-helices |  | conserved |  |  |
|  |  | **0928con3** |  | **61.88** |  | **53.55** |  | **61.46** |  | **nt-11 “T”**  **nt-16 “T”** |  | **conserved** |  | **conserved** |  | **not formed** |  | **not formed** |  | **pseudogene** |
|  |  | **0928con4** |  | **56.50** |  | **48.39** |  | **56.10** |  | **nt-11 “T”** |  | **nt-7 “T”**  **nt-9 “A”** |  | **nt-4 “A”** |  | **not formed** |  | **conserved** |  | **pseudogene** |
|  |  |  |  |  |  |  |  |  |  |  |  |  |  |  |  |  |  |  |  |  |
| Sar |  | 1213con1 |  | 67.71 |  | 58.06 |  | 66.83 |  | conserved |  | conserved |  | conserved |  | four-helices |  | conserved |  |  |
|  |  | **1213con2** |  | **57.85** |  | **53.55** |  | **66.83** |  | **nt-11 “T”** |  | **nt-7 “T”** |  | **conserved** |  | **four-helices** |  | **not formed** |  | **pseudogene** |
|  |  | **1213con3** |  | **57.87** |  | **54.19** |  | **63.55** |  | **nt-11 “T”**  **nt-16 “T”** |  | **nt-14 “T”** |  | **conserved** |  | **not formed** |  | **not formed** |  | **pseudogene** |
|  |  | 1213con4 |  | 62.50 |  | 57.42 |  | 68.87 |  | conserved |  | conserved |  | conserved |  | four-helices* |  | conserved |  |  |
|  |  |  |  |  |  |  |  |  |  |  |  |  |  |  |  |  |  |  |  |  |
| Umbubu |  | 0854con1 |  | 67.71 |  | 58.06 |  | 67.32 |  | conserved |  | conserved |  | conserved |  | four-helices |  | conserved |  |  |
|  |  | **0854con2** |  | **57.85** |  | **54.84** |  | **63.90** |  | **nt-11 “T”** |  | **conserved** |  | **conserved** |  | **not formed** |  | **not formed** |  | **pseudogene** |
|  |  | 0854con3 |  | 61.11 |  | 56.13 |  | 69.81 |  | nt-16 “T” |  | conserved |  | conserved |  | four-helices |  | conserved |  |  |
|  |  |  |  |  |  |  |  |  |  |  |  |  |  |  |  |  |  |  |  |  |
| Butuhan |  | 1074con1 |  | 62.33 |  | 57.14 |  | 70.97 |  | conserved |  | conserved |  | conserved |  | four-helices |  | conserved |  |  |
|  |  |  |  |  |  |  |  |  |  |  |  |  |  |  |  |  |  |  |  |  |

**▲** Name of ITS type is based on the ITC accession number and is used in phylogenetic trees. The consensus sequences of the ITS region in diploid and triploid species with polymorphic ITS are labeled as “con + number”.

***** Putative pseudogenic character of the ITS sequences was estimated in *silico* (see Materials and Methods).
