# Supplementary material for: The ITS1-5.8S-ITS2 Sequence Region in the Musaceae: Structure, Diversity and Use in Molecular Phylogeny
Source: PLoS One. 2011 Mar 22;6(3):e17863. doi: 10.1371/journal.pone.0017863 (PMC3062550; doi:10.1371/journal.pone.0017863)
Supplement: Table S3 — Table showing genome composition of hybrid clones analyzed in this study and corresponding ITS sequence types identified after NJ and BI analysis. (DOC) [file pone.0017863.s007.doc]

**Table S3. P**reviously described genome composition of hybrid clones and corresponding ITS sequence types identified in this study

| **Genome composition**  **(MGIS database)** |  | **Accession name** |  | **Name of ITS type▲** |  | **ITS nucleotide sequence type** |  | **Position of putative pseudogene in the phylogenetic tree** |
| --- | --- | --- | --- | --- | --- | --- | --- | --- |
|  |  |  |  |
|  |  |  |  |  |  |  |  |  |
| AAA |  | Pisang Bakar |  | 1064con1 |  | A |  |  |
|  |  |  |  | 1064con2 |  | A |  |  |
|  |  |  |  | 1064con3 |  | A |  |  |
|  |  |  |  |  |  |  |  |  |
| AAA |  | Grande Naine |  | NEU0172con1 |  | A |  |  |
|  |  |  |  | NEU0172con2 |  | A |  |  |
|  |  |  |  | NEU0172con3 |  | A |  |  |
|  |  |  |  | NEU0172con4 |  | A |  |  |
|  |  |  |  |  |  |  |  |  |
| AAA |  | Gros Michel |  | 0484con1 |  | A |  |  |
|  |  |  |  | 0484con2 |  | A |  |  |
|  |  |  |  |  |  |  |  |  |
| AAA |  | Red Dacca |  | 0575con1 |  | A |  |  |
|  |  |  |  | 0575con2 |  | A |  |  |
|  |  |  |  | 0575con3 |  | A |  |  |
|  |  |  |  |  |  |  |  |  |
| AAA |  | Pisang Kayu |  | **0420con1** |  | **pseudogenic*** |  | **Eumusa/Rhodochlamys clade**  **(see Supplementary 6)** |
|  |  |  |  | 0420con2 |  | A |  |  |
|  |  |  |  | **0420con3** |  | **pseudogenic*** |  | **subclade A (see Supplementary 6)** |
|  |  |  |  | 0420con4 |  | A |  |  |
|  |  |  |  |  |  |  |  |  |
| AAA |  | Gran Enano |  | 1256con1 |  | A |  |  |
|  |  |  |  | **1256con2** |  | **pseudogenic*** |  | **Eumusa/Rhodochlamys clade**  **(see Supplementary 6)** |
|  |  |  |  | 1256con3 |  | A |  |  |
|  |  |  |  | 1256con4 |  | A |  |  |
|  |  |  |  |  |  |  |  |  |
| AAA |  | Hochuchu |  | 0549con1 |  | A |  |  |
|  |  |  |  | 0549con2 |  | A |  |  |
|  |  |  |  | **0549con3** |  | **pseudogenic*** |  | **subclade A (see Supplementary 6)** |
|  |  |  |  |  |  |  |  |  |
| AAA |  | Not named  M. paradisiaca |  | 0089con1 |  | A |  |  |
|  |  |  |  | **0089con2** |  | **pseudogenic*** |  | **Eumusa/Rhodochlamys clade**  **(see Supplementary 6)** |
|  |  |  |  | **0089con3** |  | **pseudogenic*** |  | **subclade A (see Supplementary 6)** |
|  |  |  |  | **0089con4** |  | **pseudogenic*** |  | **Eumusa/Rhodochlamys clade**  **(see Supplementary 6)** |
|  |  |  |  |  |  |  |  |  |
| AAA |  | Not named  M. paradisiaca x |  | 0544con1 |  | A |  |  |
|  |  |  |  | 0544con2 |  | A |  |  |
|  |  |  |  | **0544con3** |  | **pseudogenic*** |  | **subclade A (see Supplementary 6)** |
|  |  |  |  | **0544con4** |  | **pseudogenic*** |  | **subclade A (see Supplementary 6)** |
|  |  |  |  |  |  |  |  |  |
| AAA |  | Novaria |  | 1329con1 |  | A |  |  |
|  |  |  |  | 1329con2 |  | A |  |  |
|  |  |  |  | 1329con3 |  | A |  |  |
|  |  |  |  |  |  |  |  |  |
| AAB |  | Maritú |  | **0639con1** |  | **pseudogenic*** |  | **subclade A (see Supplementary 6)** |
|  |  |  |  | 0639con2 |  | A |  |  |
|  |  |  |  |  |  |  |  |  |
| AAB |  | Obino l'Ewai |  | **0109con1** |  | **pseudogenic*** |  | **subclade B (see Supplementary 6)** |
|  |  |  |  | 0109con2 |  | B |  |  |
|  |  |  |  | **0109con3** |  | **pseudogenic*** |  | **subclade A (see Supplementary 6)** |
|  |  |  |  |  |  |  |  |  |
| AAB |  | 3 Hands Planty |  | **1132con1** |  | **pseudogenic*** |  | **subclade B (see Supplementary 6)** |
|  |  |  |  | **1132con2** |  | **pseudogenic*** |  | **subclade A (see Supplementary 6)** |
|  |  |  |  | 1132con3 |  | A |  |  |
|  |  |  |  | 1132con4 |  | A |  |  |
|  |  |  |  |  |  |  |  |  |
| AAB |  | Popoulou (CMR) |  | 1135con1 |  | A |  |  |
|  |  |  |  | **1135con2** |  | **pseudogenic*** |  | **subclade A (see Supplementary 6)** |
|  |  |  |  | 1135con3 |  | B |  |  |
|  |  |  |  | **1135con4** |  | **pseudogenic*** |  | **subclade B (see Supplementary 6)** |
|  |  |  |  |  |  |  |  |  |
| ABB |  | Cachaco Enano |  | 0632con1 |  | B |  |  |
|  |  |  |  | 0632con2 |  | B |  |  |
|  |  |  |  |  |  |  |  |  |
| ABB |  | Cachaco |  | **0643con1** |  | **pseudogenic*** |  | **subclade B (see Supplementary 6)** |
|  |  |  |  | **0643con2** |  | **pseudogenic*** |  | **subclade B (see Supplementary 6)** |
|  |  |  |  | **0643con3** |  | **pseudogenic*** |  | **subclade A (see Supplementary 6)** |
|  |  |  |  | 0643con4 |  | A |  |  |
|  |  |  |  | 0643con5 |  | A |  |  |
|  |  |  |  | **0643con6** |  | **pseudogenic*** |  | **Eumusa/Rhodochlamys clade**  **(see Supplementary 6)** |
|  |  |  |  |  |  |  |  |  |
|  |  |  |  |  |  |  |  |  |
|  | | | | | | | | |
|  | | | | | | | | |

| **Table S3. Continued** | | | | | | | | |
| --- | --- | --- | --- | --- | --- | --- | --- | --- |
| **Genome composition**  **(MGIS database)** |  | **Accession name** |  | **Name of ITS type▲** |  | **ITS nucleotide sequence type** |  | **Position of putative pseudogene in the tree** |
|  |  |
|  |  |  |  |  |  |  |  |  |
| ABB |  | Dole |  | **0767con1** |  | **pseudogenic*** |  | **subclade B (see Supplementary 6)** |
|  |  |  |  | **0767con2** |  | **pseudogenic*** |  | **subclade A (see Supplementary 6)** |
|  |  |  |  | 0767con3 |  | B |  |  |
|  |  |  |  | 0767con4 |  | A |  |  |
|  |  |  |  | 0767con5 |  | A |  |  |
|  |  |  |  |  |  |  |  |  |
| ABB |  | Kivuvu |  | 0157con1 |  | B |  |  |
|  |  |  |  | 0157con2 |  | B |  |  |
|  |  |  |  |  |  |  |  |  |
| ABB |  | Silver Bluggoe |  | 0364con1 |  | A |  |  |
|  |  |  |  | 0364con2 |  | B |  |  |
|  |  |  |  |  |  |  |  |  |
| ABB |  | Saba |  | 1138con1 |  | B |  |  |
|  |  |  |  | 1138con2 |  | A |  |  |
|  |  |  |  | 1138con3 |  | A |  |  |
|  |  |  |  |  |  |  |  |  |
| ABB |  | Pelipita |  | 0472con1 |  | B |  |  |
|  |  |  |  | 0472con2 |  | A |  |  |
|  |  |  |  | **0472con3** |  | **pseudogenic*** |  | **subclade A (see Supplementary 6)** |
|  |  |  |  |  |  |  |  |  |
| AS |  | Ato |  | 0820con1 |  | S |  |  |
|  |  |  |  | 0820con2 |  | A |  |  |
|  |  |  |  | 0820con3 |  | S |  |  |
|  |  |  |  |  |  |  |  |  |
| AS |  | Tonton Kepa |  | 0822con1 |  | S |  |  |
|  |  |  |  |  |  |  |  |  |
| AS |  | Ungota |  | 0954con1 |  | S |  |  |
|  |  |  |  | 0954con2 |  | A |  |  |
|  |  |  |  |  |  |  |  |  |
| AxS |  | M. acuminata ssp. x M. schizocarpa |  | 1014con1 |  | S |  |  |
|  |  |  |  |  |  |  |  |  |
|  |  |  |  |  |  |  |  |  |
| AxT |  | M. jackeyi |  | 0851con1 |  | T |  |  |
|  |  |  |  | 0851con2 |  | A |  |  |
|  |  |  |  | **0851con3** |  | **pseudogenic*** |  | **Australimusa/Callimusa clade** |
|  |  |  |  |  |  |  |  |  |
| AxT |  | Kabulupusa |  | 0928con1 |  | T |  |  |
|  |  |  |  | 0928con2 |  | A |  |  |
|  |  |  |  | **0928con3** |  | **pseudogenic*** |  | **Australimusa/Callimusa clade** |
|  |  |  |  | **0928con4** |  | **pseudogenic*** |  | **Australimusa/Callimusa clade** |
|  |  |  |  |  |  |  |  |  |
| AxT |  | Sar |  | 1213con1 |  | T |  |  |
|  |  |  |  | **1213con2** |  | **pseudogenic*** |  | **Australimusa/Callimusa clade** |
|  |  |  |  | **1213con3** |  | **pseudogenic*** |  |  |
|  |  |  |  | 1213con4 |  | A |  |  |
|  |  |  |  |  |  |  |  |  |
| AxT |  | Umbubu |  | 0854con1 |  | T |  |  |
|  |  |  |  | **0854con2** |  | **pseudogenic*** |  | **Australimusa/Callimusa clade** |
|  |  |  |  | 0854con3 |  | A |  |  |
|  |  |  |  |  |  |  |  |  |
| BxT |  | Butuhan |  | 1074con1 |  | B |  |  |
|  |  |  |  |  |  |  |  |  |

**▲** Name of ITS type is based on the ITC accession number and is used in phylogenetic trees. The consensus sequences of the ITS region in diploid and triploid species with polymorphic ITS are labeled as “con + number”.

***** Putative pseudogenic character of the ITS sequences was estimated in *silico* (see Materials and Methods).
