# Supplementary material for: The ITS1-5.8S-ITS2 Sequence Region in the Musaceae: Structure, Diversity and Use in Molecular Phylogeny
Source: PLoS One. 2011 Mar 22;6(3):e17863. doi: 10.1371/journal.pone.0017863 (PMC3062550; doi:10.1371/journal.pone.0017863)
Supplement: Table S4 — Representatives of the order Zingiberales. (DOC) [file pone.0017863.s008.doc]

**Table S4**: Representatives of the order Zingiberales

| **Family** |  | **Genus** |  | **Species** |  | **GenBank code** |  | **Reference** |
| --- | --- | --- | --- | --- | --- | --- | --- | --- |
| **Costaceae** |  |  |  |  |  |  |  |  |
|  |  | Costus |  | afer |  | AY994744 |  | Specht 2006 |
|  |  |  |  | allenii |  | AY041043 |  | Specht et al. 2001 |
|  |  |  |  | amazonicus |  | AY041032 |  | Specht et al. 2001 |
|  |  |  |  | amazonicus krukovii |  | AY972879 |  | Kay et al. 2005 |
|  |  |  |  | arabicus |  | AY041034 |  | Specht et al. 2001 |
|  |  |  |  | asplundii |  | AY972885 |  | Kay et al. 2005 |
|  |  |  |  | barbatus |  | AY994741 |  | Specht 2006 |
|  |  |  |  | bracteatus |  | AY972892 |  | Kay et al. 2005 |
|  |  |  |  | chartaceus |  | AY994719 |  | Specht 2006 |
|  |  |  |  | clavigerA |  | AY994740 |  | Specht 2006 |
|  |  |  |  | clavigerB |  | AY972882 |  | Kay et al. 2005 |
|  |  |  |  | comosus comosus |  | AY972924 |  | Kay et al. 2005 |
|  |  |  |  | deistelii |  | AY994752 |  | Specht 2006 |
|  |  |  |  | dirzoi |  | AY972930 |  | Kay et al. 2005 |
|  |  |  |  | erythrocoryne |  | AY972886 |  | Kay et al. 2005 |
|  |  |  |  | erythrophyllus |  | AY972912 |  | Kay et al. 2005 |
|  |  |  |  | erythrothyrsus |  | AY972889 |  | Kay et al. 2005 |
|  |  |  |  | gabonensis |  | AY994747 |  | Specht 2006 |
|  |  |  |  | globosus |  | AF434894 |  | Johansen 2005 |
|  |  |  |  | guanaiensis guanaiensis |  | AY972883 |  | Kay et al. 2005 |
|  |  |  |  | guanaiensis macrostrobilus |  | AY972917 |  | Kay et al. 2005 |
|  |  |  |  | guanaiensis tarmicus |  | GQ294460 |  | Kay and Yost 2009 |
|  |  |  |  | laevis |  | AY972922 |  | Kay et al. 2005 |
|  |  |  |  | lasius |  | AY972893 |  | Kay et al. 2005 |
|  |  |  |  | lateriflorus |  | AY994734 |  | Specht 2006 |
|  |  |  |  | laterifolius |  | AY972940 |  | Kay et al. 2005 |
|  |  |  |  | letestui |  | AY994733 |  | Specht 2006 |
|  |  |  |  | letestui |  | AY972939 |  | Kay et al. 2005 |
|  |  |  |  | lima lima |  | AY972926 |  | Kay et al. 2005 |
|  |  |  |  | lima scabremarginatus |  | AY972925 |  | Kay et al. 2005 |
|  |  |  |  | longebracteolatus |  | AY972887 |  | Kay et al. 2005 |
|  |  |  |  | maculatus |  | AY994731 |  | Specht 2006 |
|  |  |  |  | malortieanus |  | AY994732 |  | Specht 2006 |
|  |  |  |  | megalobractea |  | AY994730 |  | Specht 2006 |
|  |  |  |  | montanus |  | AY972929 |  | Kay et al. 2005 |
|  |  |  |  | mosaicus |  | AY994728 |  | Specht 2006 |
|  |  |  |  | nitidus |  | GQ294458 |  | Kay and Yost 2009 |
|  |  |  |  | osae |  | AY972927 |  | Kay et al. 2005 |
|  |  |  |  | phaeotrichus |  | AY994721 |  | Specht 2006 |
|  |  |  |  | pictus |  | AY041033 |  | Specht et al. 2001 |
|  |  |  |  | plicatus |  | AY994725 |  | Specht 2006 |
|  |  |  |  | productus |  | AY972895 |  | Kay et al. 2005 |
|  |  |  |  | pulverulentus |  | AY972897 |  | Kay et al. 2005 |
|  |  |  |  | ricus |  | GQ294461 |  | Kay and Yost 2009 |
|  |  |  |  | scaber |  | AY972902 |  | Kay et al. 2005 |
|  |  |  |  | spicatus |  | AY972903 |  | Kay et al. 2005 |
|  |  |  |  | spiralisA |  | AY972915 |  | Kay et al. 2005 |
|  |  |  |  | spiralisB |  | AY972914 |  | Kay et al. 2005 |
|  |  |  |  | stenophyllusA |  | AY994720 |  | Specht 2006 |
|  |  |  |  | stenophyllusB |  | AY972931 |  | Kay et al. 2005 |
|  |  |  |  | talbotii |  | AY972937 |  | Kay et al. 2005 |
|  |  |  |  | tappenbeckianus |  | AY994715 |  | Specht 2006 |
|  |  |  |  | vargasii |  | GQ294462 |  | Kay and Yost 2009 |
|  |  |  |  | varzearum |  | AY994722 |  | Specht 2006 |
|  |  |  |  | villosissimus |  | AY994713 |  | Specht 2006 |
|  |  |  |  | vinosus |  | AY972923 |  | Kay et al. 2005 |
|  |  |  |  | wilsonii |  | AY972921 |  | Kay et al. 2005 |
|  |  |  |  | woodsonii |  | AY994712 |  | Specht 2006 |
|  |  |  |  | zingiberoides |  | AY972910 |  | Kay et al. 2005 |
|  |  |  |  |  |  |  |  |  |
| **Strelitziaceae** |  |  |  |  |  |  |  |  |
|  |  | Strelitzia |  | alba |  | AF434902 |  | Johansen 2005 |
|  |  |  |  |  |  |  |  |  |
| **Heliconiaceae** |  |  |  |  |  |  |  |  |
|  |  | Heliconia |  | irrasa |  | AY673071 |  | Prince and Kress 2006 |
|  |  |  |  | rostrata |  | AF434898 |  | Johansen 2005 |
|  |  |  |  | solomonensis |  | AF434899 |  | Johansen 2005 |
|  |  |  |  |  |  |  |  |  |

**Table S4**: Continued

| **Family** |  | **Genus** |  | **Species** |  | **GenBank code** |  | **Reference** |
| --- | --- | --- | --- | --- | --- | --- | --- | --- |
| **Lowiaceae** |  |  |  |  |  |  |  |  |
|  |  | Orchidantha |  | borneensisA |  | AF434877 |  | Johansen 2005 |
|  |  |  |  | borneensisB |  | AF434876 |  | Johansen 2005 |
|  |  |  |  | chinensis |  | AF434878 |  | Johansen 2005 |
|  |  |  |  | fimbriata |  | AF434879 |  | Johansen 2005 |
|  |  |  |  | grandiflora |  | AF434880 |  | Johansen 2005 |
|  |  |  |  | holttumii |  | AF434881 |  | Johansen 2005 |
|  |  |  |  | inouei |  | AF434882 |  | Johansen 2005 |
|  |  |  |  | longiflora |  | AF434883 |  | Johansen 2005 |
|  |  |  |  | maxillarioides |  | AF434884 |  | Johansen 2005 |
|  |  |  |  | quadricolor |  | AF434885 |  | Johansen 2005 |
|  |  |  |  | sabahensis |  | AF434886 |  | Johansen 2005 |
|  |  |  |  | siamensis |  | AF434887 |  | Johansen 2005 |
|  |  |  |  | suratii |  | AF434890 |  | Johansen 2005 |
|  |  |  |  |  |  |  |  |  |
